# Different psychological interventions for perinatal depression: a systematic review and meta-analysis of randomized controlled trials

**DOI:** 10.1186/s12888-025-07462-3

**Published:** 2025-10-14

**Authors:** Guangshun Hua, Keling Yue, Yu Zhu, Fuchao Yang, Mijuan Zhou

**Affiliations:** Zigong first peopleʼs hospital · Zigong academy of medical, Zigong, China

**Keywords:** Perinatal depression, Psychotherapy, Meta-analysis

## Abstract

**Background:**

Psychological interventions are the preferred treatment for women with perinatal depression, but there is a lack of comprehensive meta-analyses evaluating their effectiveness and the impact of related variables.

**Objective:**

We performed a systematic review and meta-analysis to assess the efficacy of psychological interventions for perinatal depression and to examine the influence of associated variables.

**Methods:**

We systematically searched the Ovid platform, covering the MEDLINE, EMBASE, PsycINFO, and Web of Science databases from their inception to March 11, 2024.

**Results:**

We screened 5,827 articles, and 33 were included in a random-effects meta-analysis. Compared to the control groups, psychological interventions showed a moderate pooled effect size (SMD: -0.65; 95% CI: -0.87 to -0.43, moderate certainty of evidence). Subgroup analyses suggested that individualized interventions tended to be more effective than group‑based approaches, although this difference may be influenced by other factors such as intervention duration or setting. Additionally, interventions delivered in non‑clinical settings (e.g., participants’ homes or community centers) maybe been more effective than those delivered in clinical settings. In the included studies, non-specialist therapists who received professional training appeared to achieve treatment outcomes for perinatal depression that were broadly similar to those reported for specialist therapists. Variability in the effectiveness of different psychological interventions was observed, with IPT showing a tendency toward a larger effect size compared to CBT.

**Limitations:**

This study has high heterogeneity; the limited number of studies on MBI, BA, and PST may affect the accuracy of the meta-analysis results.

**Conclusions:**

IPT, CBT, MBI, and BA have been shown to effectively alleviate perinatal depression, while PST did not demonstrate significant efficacy. IPT appeared to be more effective than the other interventions, although direct head-to-head comparisons were not conducted. Additionally, personalized interventions in non-clinical settings may be more beneficial. Furthermore, a trained non-mental health therapist may also achieve positive outcomes when delivering psychological interventions.

**Supplementary Information:**

The online version contains supplementary material available at 10.1186/s12888-025-07462-3.

## Introduction

Perinatal depression (PPD) is recognized as a significant symptom of major depressive disorder, usually appearing during pregnancy or within a few weeks after birth. In both clinical and research settings, this period is often extended to include the entire first year postpartum [1]. The worldwide prevalence of perinatal depression is estimated to range from 13% to 30%, with higher rates seen in middle- and low-income countries. Although the exact causes are not fully understood, prevailing theories suggest that increased sensitivity to hormonal changes during the perinatal period, dysregulation of the hypothalamic-pituitary-adrenal axis, and immune system changes play major roles in its development. Identified risk factors include a history of psychiatric conditions, lack of social support, intimate partner violence, low socioeconomic status, and health issues affecting the mother and child [2]. Despite the common belief that perinatal depression may go away on its own, many cases last for months or even longer, especially without prompt treatment. Besides the typical symptoms of ongoing depression, fatigue, sadness, and guilt, women with perinatal depression face a higher risk of negative health outcomes during the perinatal period. The condition has been linked to pregnancy complications like preeclampsia and gestational diabetes. Additionally, women diagnosed with perinatal depression show an increased risk of death [3], especially from unnatural causes such as suicide, with the highest risk occurring within the first year after diagnosis. Importantly, this risk seems to be independent of any prior psychiatric history or family history.

Currently, various screening tools for perinatal depression (e.g., Edinburgh Postnatal Depression Scale) are widely used in clinical settings to evaluate women showing symptoms. However, stigma associated with perinatal depression and the lack of cognitive symptoms may hinder further assessment [4]. A range of interventions has been implemented during the perinatal period to treat or prevent depression, including pharmacotherapy, dietary changes, and psychotherapy. On August 4, 2023, the U.S. Food and Drug Administration (FDA) approved zuranolone, the first oral medication for postpartum depression. Zuranolone, a neuroactive steroid and γ-aminobutyric acid receptor–positive allosteric modulator, is seen as a new, potentially rapid-acting oral treatment for postpartum depression (PPD) [5]. However, it seems unlikely that any single medication will significantly reduce the overall burden of perinatal depression. For most mothers, pharmacotherapy is not recommended as the initial treatment. The most effective approach appears to involve strategies that target the psychosocial factors contributing to perinatal depression. Additionally, dietary interventions for perinatal depression have shown limited success. Despite the common use of polyunsaturated fatty acids (PUFAs) and trace elements in pregnant women’s diets, they do not seem to significantly alleviate perinatal depression. However, daily intake of 1800–3500 International Units of vitamin D shows some potential [6].

Women with postpartum depression often favor psychotherapy as the primary treatment due to its proven effectiveness and lack of medication-related risks [7–9]. CBT employs structured, skills-based sessions to change maladaptive thoughts and behaviors and is effective in individual, group, and digital formats [10]; IPT addresses interpersonal stressors (role transitions, grief, relationship issues) in brief, weekly sessions [11]. Problem-Solving Therapy (PST), Behavioral Activation (BA), and Mindfulness-Based Interventions (MBIs) show beneficial but more varied results: PST offers brief, structured problem-definition and solution strategies; BA enhances engagement in rewarding activities to reduce avoidance; MBIs foster present-moment, nonjudgmental awareness to decrease rumination and anxiety [12–14]. Overall, CBT and IPT have the strongest and most consistent evidence for perinatal care, while PST, BA, and MBIs are promising and warrant further rigorous study. Existing meta-analyses mainly focus on Cognitive Behavioral Therapy (CBT) and Interpersonal Therapy (IPT), with limited attention to other psychotherapy types like PST, BA, and MBIs.

So far, no review has thoroughly summarized the effects of psychotherapeutic interventions, including their implications for clinical decision-making by pregnant and postpartum women and their healthcare providers. Therefore, this systematic review and meta-analysis aim to evaluate the effectiveness of published psychotherapeutic approaches for perinatal depression and related factors. We also intend to identify the design features and components of intervention programs associated with better treatment outcomes. Our goal is to develop more targeted psychotherapeutic strategies for treating perinatal depression and provide valuable insights for researchers, perinatal care providers, and other healthcare professionals.

## Methods

### Protocol registration

The PRISMA guidelines [15] and checklists were followed (Appendix S1). The study protocol for this systematic review and meta-analysis was published in the PROSPERO database (CRD42024526766).

### Search strategy

A systematic search of electronic databases (MEDLINE, Embase, PsycINFO, and Cochrane Library [Cochrane Central Register of Controlled Trials]) was conducted via Ovid up to March 11, 2024. We cross-checked the reference lists of key reviews. The search strategy was developed by combining keywords and Medical Subject Headings (MeSH) terms (Appendix S2), covering three concepts: pregnancy and/or postpartum period, depression, and psychological intervention.

### Eligibility criteria

Studies were included if they (1) involved psychological interventions for depression during pregnancy or within one year postpartum, (2) assessed changes in depression symptoms using standardized measurement tools, (3) were randomized controlled trials (RCTs), (4) and were published in English due to feasibility and the fact that most high-quality RCTs are published in English (Crutchfield and Enderson, 1989). Studies were excluded if (1) women had other perinatal mental disorders or comorbidities (e.g., OCD or personality disorders), (2) they were reviews, systematic evaluations, meta-analyses, or animal studies, or (3) they evaluated therapeutic interventions delivered via a mobile app on smartphones or tablets. Additionally, studies were excluded if they involved participants’ use of antidepressants during the perinatal period.

Two reviewers independently screened the title and abstract. We resolved disagreements through discussion, consulting a third reviewer if necessary.

### Data extraction

Two researchers independently collected the data using a standardized form, which was piloted with five randomly selected studies to ensure comprehensive data capture. We verified duplicate data with Excel, and any disagreements were resolved by a third-party reviewer. The following information was extracted: (1) study characteristics (author, publication year, region and setting, sample size, intervention implementer, duration of treatment, intervention type, funding source, follow-up and treatment duration, and treatment strategy); (2) patient characteristics (age); (3) outcome data (mean and standard deviation of results for all continuous point-in-time scores). We prioritized outcomes based on intention-to-treat analysis over per-protocol analysis. When ITT analysis was unavailable for most continuous outcomes, we used data from the conforming protocol analysis. Studies with missing data were excluded.

### Risk-of‐bias assessment

Two reviewers independently assessed the risk of bias; if they disagreed, a third reviewer discussed the issue to reach a consensus. They evaluated the methodological risk of bias for eligible studies using the Cochrane Risk of Bias 2 scale for RCTs [16]. Each aspect—such as random-sequence generation, allocation concealment, blinding, missing outcome data, and selective reporting—was rated as low, some concerns, or high risk. Publication bias was visually inspected using funnel plots created with RevMan 5.4 and was also statistically tested using the Egger test [17].

### Data analysis

RevMan 5.4 software was used to assess heterogeneity, combine data, and generate forest and funnel plots for the studies included. Effect size was measured using the standardized mean difference (SMD) and 95% confidence interval (CI), as many studies employed different rating instruments [e.g., Edinburgh Postnatal Depression Scale (EPDS), Patient Health Questionnaire (PHQ), Beck Depression Inventory (BDI), and Hamilton Depression Scale (HAMD)]. |SMD| < 0.5 indicates a small effect size, 0.5 ≤ |SMD| < 0.8 indicates a medium effect size, and |SMD| ≥ 0.8 indicates a large effect size. I^2^ values were calculated to determine heterogeneity, with 25% representing low, 50% moderate, and 75% high heterogeneity [18].

Subgroup analyses were used to explore relevant variables and assess the effectiveness and differences between various forms of psychological interventions for perinatal depression. These variables included study characteristics such as participant mean age, country, intervention duration, and evaluation standards, as well as components of the intervention like mode of intervention (face-to-face or telemedicine), professional support (professional therapist or non-professional therapist), setting (medical or non-medical environment), intervention type (group versus individual, including women-only or women plus partners), intervention period (gestation or postnatal), and intervention category (CBT, IPT, MBI, BA, PST). We conducted a multiple meta-regression analysis to explore sources of heterogeneity by including study-level covariates such as risk of bias (high, some, low), mean age, setting (medical or non-medical environment), intervention method, duration, evaluation standards, income level (high-income or lower middle-income countries), intervention type (group or individual), intervention period (gestation or postnatal), professional support (professional therapist or non-professional therapist), mode of intervention (face-to-face or telemedicine), year of publication, analysis type (ITT or PP), and intervention category (CBT, IPT, MBI, BA, PST). Statistical significance was defined as two-sided *P* < 0.05 [19]. Finally, we performed sensitivity analyses to evaluate the impact of risk of bias, and the Grading of Recommendations, Assessment, Development, and Evaluations (GRADE) methodology was also employed to assess the overall quality of the key results.

## Results

### Study selection

**Fig. 1:**
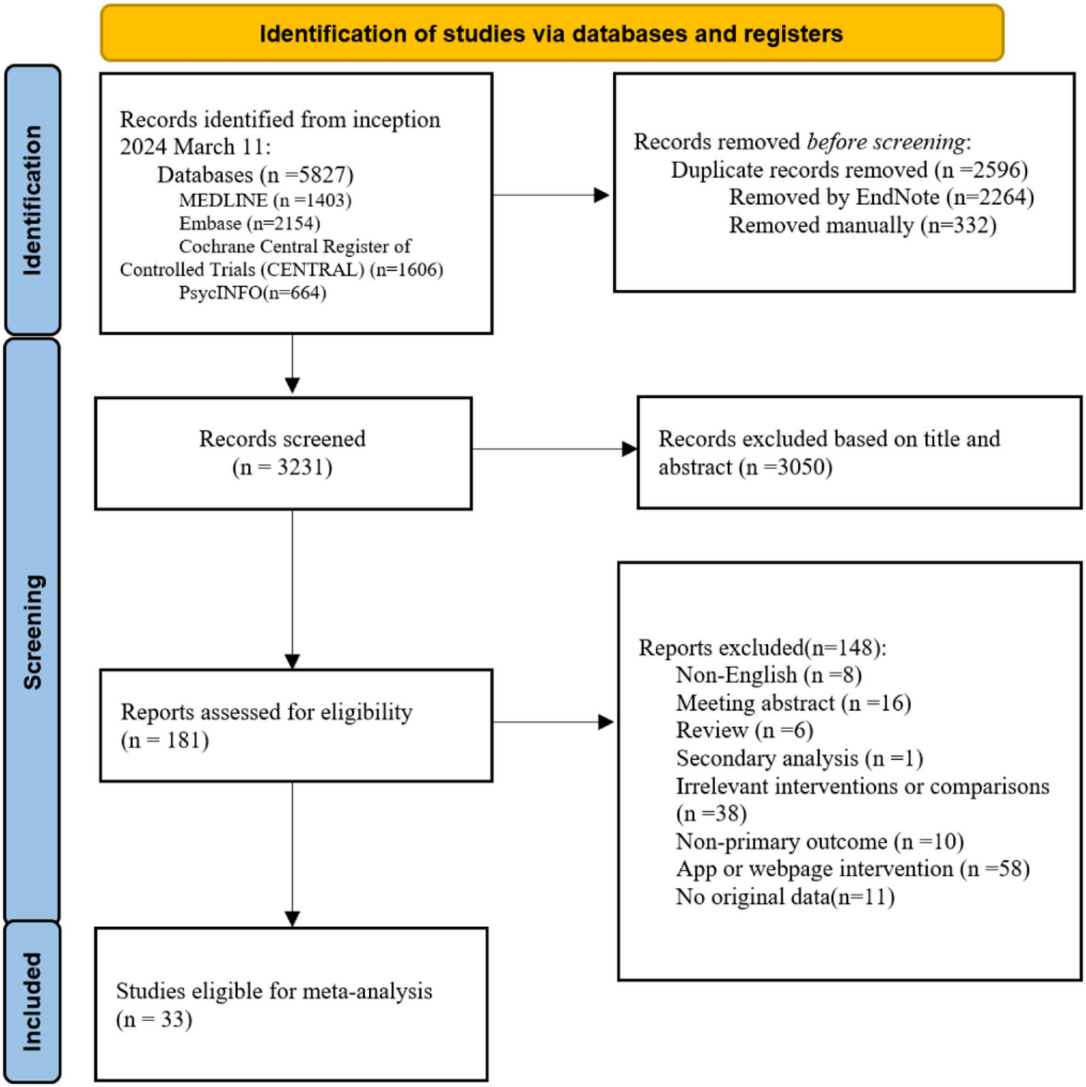
Literature screening flowchart

Our search identified 5,827 potentially relevant articles. After removing 2,596 duplicates, 3,050 studies were screened based on their titles and abstracts, with 181 studies selected for full-text review. Ultimately, 33 studies met the inclusion criteria (Fig. 1), including five studies on online-based interventions and 27 studies on face-to-face psychological interventions.

PRISMA 2020 flow diagram for new systematic reviews, which include searches of databases, registers, and other sources.

### Characteristics of the included studies

The 33 studies enrolled a total of 4,570 participants, with 2,281 receiving psychological interventions and 2,289 serving as controls. Four studies used a waitlist control group, while the remaining 29 employed active control groups. Eleven studies took place in the United States, eight in Canada, five in China, two each in the United Kingdom, Australia, and Iran, and one each in Sweden, Kenya, and Jordan. The average age of participants primarily ranged from 20 to 35 years.

All 33 studies identified depression as the primary outcome. Twenty-six studies used the Edinburgh Postnatal Depression Scale (EPDS), with most setting an inclusion cutoff of a score ≥ 10. Five studies used the Beck Depression Inventory (BDI), while the remaining two employed the Hamilton Depression Rating Scale (HAMD) and the Patient Health Questionnaire-9 (PHQ-9), respectively.

### Quality assessment

Among the 33 included studies, 6 were assessed as having a high risk of bias, 16 were considered to have some concerns about bias, and 11 were rated as having a low risk of bias (Appendix S3). The inherent challenges of blinding participants and treatment providers in psychological interventions for perinatal women often lead to deviations from intended interventions, which can increase the overall bias scores. This may impact the reliability of the outcome assessments.

### Overall association of psychotherapy with perinatal depression

**Fig. 2:**
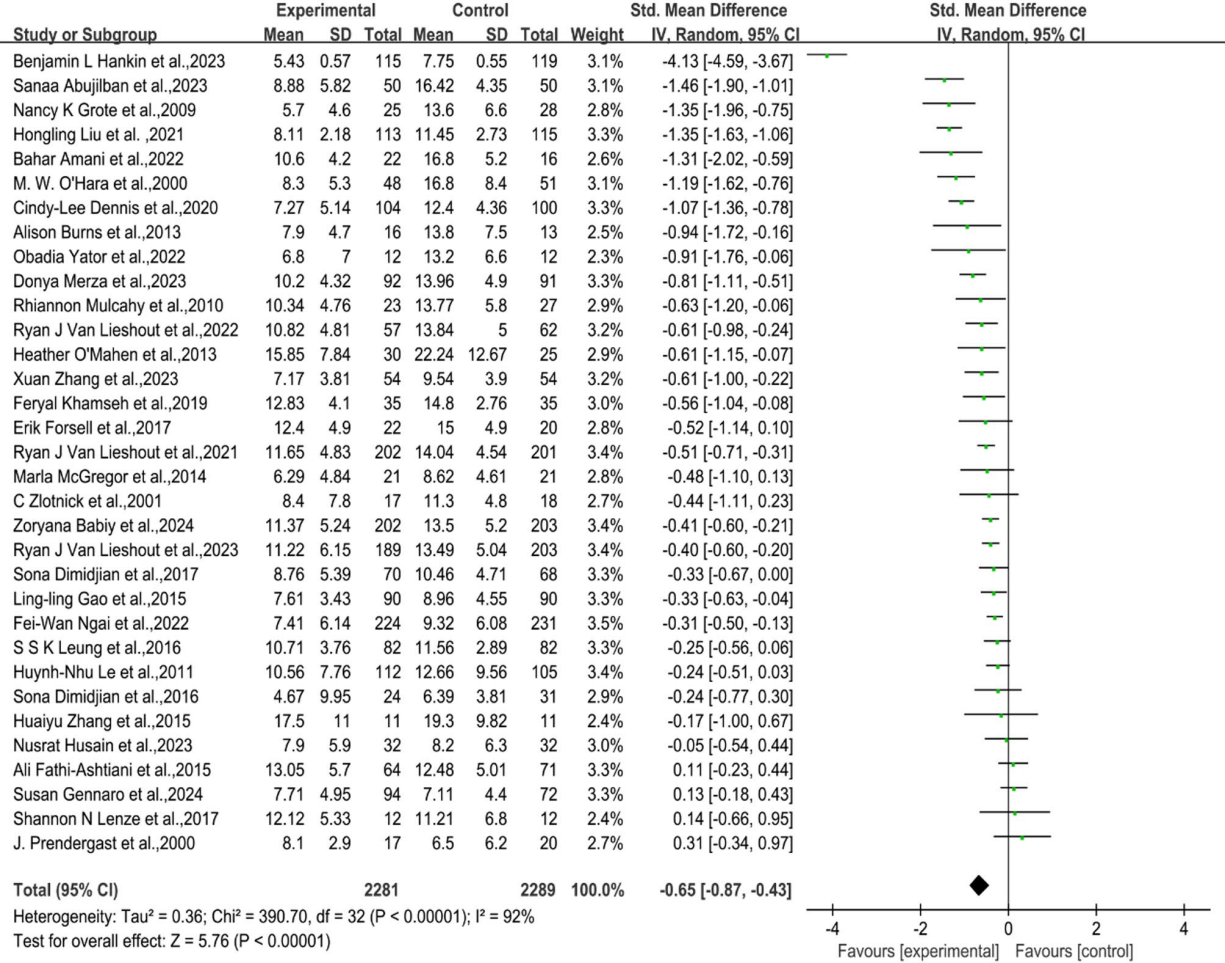
Forest plot of a randomized controlled trial testing the effectiveness of a psychological intervention for treating perinatal depression

Our meta-analysis of 33 studies on psychological interventions for perinatal depression found significant heterogeneity (*p* < 0.0001, I2 = 92%), leading to the use of a random-effects model for outcome analysis. The results showed that depression scores were lower in the psychological intervention group compared to the control group (SMD: −0.65; 95% CI: −0.87 to −0.43), with the overall effect size indicating a moderate and statistically significant difference (Fig. 2). Although the funnel plot displayed slight asymmetry, a formal statistical test (Egger’s test) confirmed the plot’s symmetry (*p* = 0.265) (Appendix S4), supporting the absence of publication bias.

A random effects model was used to generate the graphs with RevMan.RCT, a randomized controlled trial.

### Sensitivity analyses

We performed a sensitivity analysis using the leave-one-out method and compared each newly calculated effect size (95% CI) after omitting a study with the overall effect size. The results showed that each new estimate was consistent with the overall estimate, indicating the stability of the findings (Appendix S5).

### Subgroup analyses

#### Subgroup analyses of study characteristics

We conducted a subgroup analysis based on study characteristics (Table 1), revealing that studies using ITT analysis showed a smaller effect size (SMD: −0.54; 95% CI: −0.71 to −0.36). Conversely, studies with a low risk of bias (ROB) displayed a larger effect size (SMD: −0.83; 95% CI: −1.25 to −0.40). Interestingly, studies with a high ROB showed a higher effect size (SMD: −0.70; 95% CI: −1.27 to −0.12) than those with some ROB (SMD: −0.50; 95% CI: −0.74 to −0.25).

**Table 1:** Characteristics of the included studies

Study (author, year, country)	Age ± SD (years)	Duration of treatment	Treatment group(s) (*n*)	Comparison group(s) (*n*)	Baseline and assessment timepoint(s)	Outcome’ scales	Main results: SMD^1^ (95% CI)^2^
van Ravesteyn et al., 2017[20]	31.6 ± 5.2	1 Day	CBT:229	Placebo (TAU): 232	Baseline: a year of delivery ATP: 12 wk after baseline	EPDS	EPDS: −0.40 [−0.60, −0.20]
Ngai and Gao, 2022[21]	33.3 ± 3.5	6 weeks	IPT:224	Placebo (TAU):231	Baseline:12–30 wk of postpartum ATP:6 wk and 6 mo after baseline	EPDS	EPDS 6 wk: −0.31 [−0.50, −0.13] 6 mo: −0.14 [−0.32, 0.04]
Gao et al., 2015[22]	28.49 ± 2.73	2 weeks	IPT:90	Placebo (TAU): 90	Baseline: a month of delivery ATP: 2 wk after baseline	EPDS	EPDS: −0.33 [−0.63, −0.04]
Lenze and Potts, 2017 [23]	26.90 ± 5.81	9 weeks	IPT:21	Placebo (EUC): 21	Baseline:12–30 wk of GA ATP:37–39 wk of GA	EPDS	EPDS:0.14 [−0.66, 0.95]
O’Mahen et al., 2013[24]	27.40 ± 5.32	16 weeks	CBT:30	Placebo (TAU): 25	Baseline: over 24 wk of GA ATP:16 wk and 3 mo after baseline	BDI-Ⅱ	BDI-Ⅱ 16 wk: −0.61 [−1.15, −0.07] 3 mo: −0.43 [−0.97, 0.11]
Mulcahy et al., 2010[25]	32.00 ± 3.27	8 weeks	IPT:29	Placebo (TAU): 28	Baseline: a year of delivery ATP:4 w and 8 w after baseline	EPDS	EPDS 4 wk.: −0.09 [−0.64, 0.47] 8 wk: −0.63 [−1.20, −0.06]
Burns et al., 2013[26]	28.20 ± 5.0	12 weeks	CBT: 18	Placebo (UC): 18	Baseline:8–18 wk of GA ATP:15 wk and 33 wk after baseline	EPDS	EPDS 15wk: −0.94 [−1.72, −0.16] 33wk: −1.18 [−2.02, −0.34]
Dimidjian et al., 2016[27]	30.98 ± 4.08	8 weeks	MBI:43	Placebo (TAU):43	Baseline:32 wk of GA ATP:8 wk of baseline、1 mo, and 6 mo of delivery	EPDS	EPDS 8 wk: −0.24 [−0.77, 0.30] 1 mo: −0.31 [−0.87, 0.24] 6 mo: −0.33 [−0.90, 0.23]
Liu and Yang, 2021[28]	26.89 ± 4.12	6 weeks	CBT:130	Placebo (TAU): 130	Baseline: a year of delivery ATP:6 wk and 6 mo after baseline	EPDS	EPDS 6 wk: −1.35 [−1.63, −1.06] 6 mo: Not applicable (6 mo data was not reported)
Le et al., 2011[29]	25.8 ± 4.4	8 weeks	CBT: 112	Placebo (UC): 105	Baseline: 24 wk of GA ATP: during early and late pregnancy(T1),6 wk(T2)、4 mo(T3) and 12 mo of postpartum(T4)	BDI-Ⅱ	BDI-Ⅱ T1: −0.24 [−0.51, 0.03] T2: 0.04 [−0.23, 0.30] T3: 0.09 [−0.18, 0.35] T4: 0.13 [−0.14, 0.39]
Zlotnick et al., 2001[30]	23.4 ± 4.41	4 weeks	IPT:17	Placebo (TAU): 18	Baseline:20–32 wk of GA ATP:3 mo of postpartum	BDI	BDI: −0.44 [−1.11, 0.23]
Grote et al., 2009[31]	24.3 ± 5.30	8 weeks	IPT:25	Placebo (EUC): 28	Baseline:10–32 wk of GA ATP:3 mo after baseline and 6 mo of postpartum	EPDS	EPDS 3 mo: −1.35 [−1.96, −0.75] 6 mo: −1.99 [−2.66, −1.32]
Amani et al., 2021[32]	32.4 ± 4.3	9 weeks	CBT:37	Placebo (WL): 36	Baseline: a year of delivery ATP:9wk and 6 mo after baseline	EPDS	EPDS 9 wk: −1.31 [−2.02, −0.59] 6 mo: Not applicable (6 mo data was not reported)
[33]	\	6 weeks	CBT:17	Placebo (TAU): 20	Baseline: a year of delivery ATP:6 wk and 6 mo after baseline	EPDS	EPDS 6 wk:0.31 [−0.34, 0.97] 6 mo: −0.36 [−1.02, 0.29]
Hankin et al., 2023[34]	29.7 ± 5.9	24 weeks	IPT:115	Placebo (EUC): 119	Baseline: under 25 wk of GA ATP:4 wk、8 wk、12 wk、16 wk、20 wk and 24 wk after baseline	EPDS	EPDS 4 wk: −1.20 [−1.47, −0.92] 8 wk: −2.28 [−2.61, −1.95] 12 wk: −3.14 [−3.52, −2.75] 16 wk: −3.68 [−4.11, −3.26] 20 wk: −4.01 [−4.46, −3.56] 24 wk:−4.13 [−4.59, −3.67]
Dimidjian et al., 2017[35]	28.75 ± 5.67	10 weeks	BA: 86	Placebo (TUA): 77	Baseline: During pregnancy ATP: 5 wk and 10 wk after baseline 、3 mo of postpartum	PHQ-9	PHQ-9 5 wk: −0.09 [−0.44, 0.25] 10 wk: −0.33 [−0.67, 0.00] 3 mo: −0.25 [−0.59, 0.10]
McGregor et al., 2014[36]	\	6 weeks	CBT:21	Placebo (TAU):21	Baseline:20–28 wk of GA ATP:38 wk of GA and 6 wk of postpartum	EPDS	EPDS 38 wk: −0.34 [−0.95, 0.27] 6 wk: −0.48 [−1.10, 0.13]
Van Lieshout et al., 2022[37]	31.4 ± 4.9	9 weeks	CBT:70	Placebo (TAU): 71	Baseline: a year of delivery ATP: 9 wk and 6 mo after baseline	EPDS	EPDS 9 wk: −0.61 [−0.98, −0.24] 6 mo: −0.73 [−1.11, −0.36]
Khamseh et al., 2019[38]	27.80 ± 4.57	5 weeks	PST: 35	Placebo (TAU): 35	Baseline: the third trimester of pregnancy ATP:5 wk and 1 mo after baseline	BDI	BDI 5 wk: −0.56 [−1.04, −0.08] 1 mo: −0.51 [−0.98, −0.03]
Leung et al., 2016[39]	31.56 ± 3.78	6 weeks	CBT:82	Placebo (TAU): 82	Baseline: 6–8 weeks of postpartum ATP: 3 mo and 6 mo after baseline	EPDS	EPDS 3 mo: −0.25 [−0.56, 0.06] 6 mo: −0.20 [−0.51, 0.11]
Zhang et al., 2023[40]	28.54 ± 3.70	4 weeks	MBI:54	Placebo (HE): 54	Baseline:12–24 wk of GA ATP:4 wk and 15 wk after baseline	EPDS	EPDS 4 wk: −0.61 [−1.00, −0.22] 15 wk: −0.71 [−1.10, −0.32]
Husain et al., 2023[41]	30.4 ± 6.0	3 months	CBT:42	Placebo (TUA): 41	Baseline: a year of delivery ATP:3 mo and 6 mo after baseline	EPDS	EPDS 3 mo: −0.05 [−0.54, 0.44] 6 mo:0.03 [−0.47, 0.53]
Fathi-Ashtiani et al., 2015[42]	25.8 ± 3.7	8 weeks	CBT: 64	Placebo (TAU): 71	Baseline: the third trimester ATP:2 wk of postpartum	EPDS	EPDS: 0.11 [−0.23, 0.44]
O’Hara et al., 2000[43]	29.4 ± 4.9	12 weeks	IPT:60	Placebo (WL): 60	Baseline: a year of delivery ATP:4 wk、 8 wk and 12 wk after baseline	HAM-D	HAM-D 4 wk: −0.56 [−0.96, −0.16] 8 wk: −0.56 [−0.96, −0.16] 12 wk: −1.19 [−1.62, −0.76]
Gennaro et al., 2024[44]	26.34 ± 5.28	15 weeks	CBT:177	Placebo (PE): 122	Baseline: under 19 wk of GA ATP:34 wk of GA	EPDS	EPDS:0.13 [−0.18, 0.43]
[45]	23.0 ± 3.0	8 weeks	IPT:12	Placebo (WL): 12	Baseline:6–12 wk of postpartum ATP: 8 wk、16 wk and 24 wk after baseline	EPDS	EPDS 8 wk: −0.91 [−1.76, −0.06] 12 wk: −0.40 [−1.21, 0.41] 16 wk: −0.23 [−1.04, 0.57]
Zhang and Emory, 2015[46]	25.3 ± 4.6	8 weeks	MBI:34	Placebo (TAU):31	Baseline:12–31 wk of GA ATP:4 wk and 8 wk after baseline	BDI-Ⅱ	BDI-Ⅱ 4 wk:0.23 [−0.46, 0.91] 8 wk: −0.17 [−1.00, 0.67]
Babiy et al., 2024[47]	32.3 ± 4.3	1 Day	iCBT:202	Placebo (TUA): 203	Baseline: a year of delivery ATP: 12 wk after baseline	EPDS	EPDS: −0.41 [−0.60, −0.21]
Merza et al., 2023[48]	31.7 ± 4.8	9 weeks	iCBT (92)	Placebo (WL):91	Baseline: a year of delivery ATP: 9 wk and 3 mo of postpartum	EPDS	EPDS 9 wk: −0.81 [−1.11, −0.51] 3 mo: Not applicable (3 mo data was not reported)
Abujilban et al., 2024[49]	29.2 ± 5.2	1 month	iIPT (53)	Placebo (TUA):51	Baseline:24–37 wk of GA ATP: 1 mo of GA	EPDS	EPDS: −1.46 [−1.90, −1.01]
Van Lieshout et al., 2021[50]	31.5 ± 4.6	1 Day	iCBT(202)	Placebo (TUA): 201	Baseline: a year of delivery ATP: 12 wk after baseline	EPDS	EPDS: −0.51 [−0.71, −0.31]
Dennis et al., 2020[51]	\	12 weeks	iIPT (120)	Placebo (TUA):121	Baseline:2–24 wk of delivery ATP:12 wk、24 wk and 36 wk after baseline	EPDS	EPDS 12 wk: −1.07 [−1.36, −0.78] 24 wk: −1.09 [−1.38, −0.79] 36 wk: −0.59 [−0.87, −0.30]
Forsell et al., 2017[52]	31.2 ± 3.7	10 weeks	iCBT(22)	Placebo (TUA):20	Baseline:10–28 wk of GA ATP: 10 wk after baseline	EPDS	EPDS: −0.52 [−1.14, 0.10]

*GA* Gestational Age, *ATP* Assessment time point(s), *EPDS* Edinburgh Postnatal Depression Scale, *BDI* Beck’s Depression Inventory, *BDI-II* Beck’s Depression Inventory-II, *PHQ-9* Patient Health Questionnaire-9, *HAM-D* Hamilton Depression Rating Scale, *CBT* Cognitive-Behavioral Therapy, *IPT* Interpersonal Psychotherapy, *PST* Problem‑solving Training, *BA* Behavioral Activation, *MBI* Mindfulness-Based Intervention, *ICBT* Internet-Delivered Cognitive-Behavioral Therapy, *iIPT* Internet-Interpersonal Psychotherapy, *TUA* Treatment as Usual, *EUC* Enhanced Usual Care, *WL* Waitlist, *UC* Usual Care, *PE* Perinatal Education, *HE* Health Education

^1^At the study endpoint, unless otherwise specified

^2^All values are SMDs. Negative values indicate that treatment is better than control

Additionally, we conducted a subgroup analysis based on the average and median intervention durations across all studies(Table 2). Interventions lasting more than 8 weeks showed a moderate to high effect size (SMD: −0.64; 95% CI: −0.90 to −0.39) compared to the control group, while those lasting 8 weeks or less had a smaller reduction in depression (SMD: −0.45; 95% CI: −0.61 to −0.29). However, the difference between these two duration groups was not statistically significant (*p* = 0.22). In terms of age, participants with an average age between 20 and 30 years demonstrated a greater effect size compared to the control group (SMD: −0.80; 95% CI: −1.25 to −0.40). Furthermore, the effect sizes for studies using the EPDS versus non-EPDS scales, as well as those conducted in high-income versus low-to-middle-income countries, were moderate, with no significant differences between groups.

**Table 2:** Subgroup analyses based on study characteristics

Characteristic	Number of literature	Sample size intervention/control, No.	Meta-analysis		Heterogeneity		Between-group tests	
			SMD (95% CI)	*P*-value	I²%	*P*-value	Chi²	*P*-value
ITT analysis	24	1842/1846	−0.54 [−0.71, −0.36]	< 0.00001	83	< 0.00001	0.95	0.33
PP analysis	9	439/443	−0.96 [−1.78, −0.13]	0.02	96	< 0.00001		
Low ROB	11	1330/1359	−0.83 [−1.25, −0.40]	0.0001	96	< 0.00001	1.92	0.38
Some ROB Concerns	16	668/638	−0.50 [−0.74, −0.25]	< 0.0001	77	< 0.00001		
High ROB	6	283/292	−0.70 [−1.27, −0.12]	0.02	89	< 0.00001		
Intervention duration, wk								
≤ 8	19	1517/1558	−0.45 [−0.61, −0.29]	< 0.00001	75	< 0.00001	1.51	0.22
>8	14	851/815	−0.64 [−0.90, −0.39]	< 0.00001	82	< 0.00001		
Mean age, y								
20s	18	936/949	−0.80 [−1.24, −0.35]	0.004	95	< 0.00001	2.34	0.13
30s	13	1188/1219	−0.43 [−0.57, −0.30]	< 0.00001	52	0.02		
Mix	2	125/121	−0.84 [−1.40, −0.28]	0.03	65	0.09		
Standard for evaluation								
EPDS	26	1958/1976	−0.69 [−0.95, −0.42]	< 0.00001	93	< 0.00001	0.78	0.38
Non-EPDS	7	323/313	−0.52 [−0.79, −0.24]	0.0002	61	0.02		
Country								
High	24	1557/1549	−0.67 [−0.96, −0.38]	< 0.00001	93	< 0.00001	0.06	0.8
Middle and Low	9	724/740	−0.61 [−0.95, −0.27]	< 0.0004	89	< 0.00001		

Abbreviations: *ITT* Intention-to-treat, *PP* Per-protocol, *ROB* Risk of bias

### Subgroup analyses of components of the intervention programs

We also conducted a subgroup analysis based on the components of the intervention programs. Compared to both the control group and group-based interventions, individualized psychotherapy (including sessions with individual women or with women and their partners) significantly lowered depression levels (SMD: −0.99; 95% CI: −1.43 to −0.54), showing a statistically significant difference between groups (*p* = 0.006) (Table 3). Additionally, interventions carried out in non-clinical settings (such as community centers, homes, or public buildings) showed a greater effect size compared to those delivered in clinical settings (SMD: −0.85; 95% CI: −1.56 to −0.15). In contrast, there was no significant difference in effect sizes between interventions during pregnancy (SMD: −0.62; 95% CI: −1.03 to −0.21) and postpartum (SMD: −0.67; 95% CI: −0.87 to −0.47), both of which had moderate effect sizes. Further subgroup analysis revealed that IPT had significant effect sizes during both periods: (SMD: −1.27; 95% CI: −2.55 to 0.01) during pregnancy and (SMD: −0.82; 95% CI: −1.21 to −0.43) postpartum. Regarding delivery mode, telemedicine interventions (SMD: −0.78; 95% CI: −1.07 to −0.49) showed slightly higher effect sizes compared to face-to-face interventions. Notably, interventions led by trained non-professional therapists demonstrated moderate to high effect sizes (SMD: −0.80; 95% CI: −1.19 to −0.40), whereas those conducted by professional therapists showed moderate to low effect sizes. Finally, our analysis of different psychological intervention types indicated that PST did not produce a significant effect compared to the control group (*p* = 0.05), with the most significant effect size observed in IPT (SMD: −1.07; 95% CI: −1.70 to −0.44), followed by CBT, MBT, and BA.

**Table 3:** Subgroup analysis based on intervention programme components

Characteristic	Number of literature	Sample size intervention/control, No.	Meta-analysis		Heterogeneity		Between-group tests	
			SMD (95% CI)	*P*-value	I²%	*P*-value	Chi²	*P*-value
Intervention number								
Group Intervention	17	1307/1327	−0.33 [−0.47, −0.20]	< 0.00001	58	0.002	7.45	0.006
Individual Intervention	15	944/937	−0.99 [−1.43, −0.54]	< 0.0001	95	< 0.00001		
Intervening Period								
Gestation	19	1013/1004	−0.62 [−1.03, −0.21]	0.03	94	< 0.00001	0.05	0.82
Postnatal	14	1268/1285	−0.67 [−0.87, −0.47]	< 0.00001	81	< 0.00001		
Setting								
Medical Environment	12	870/856	−0.51 [−0.78, −0.23]	0.0003	86	< 0.00001	0.8	0.37
Non-medical Environment	11	552/576	−0.85 [−1.56, −0.15]	0.02	96	< 0.00001		
Professional Support								
Yes	15	885/906	−0.52 [−0.73, −0.31]	< 0.00001	75	< 0.00001	0.32	1.42
No	16	1325/1311	−0.80 [−1.19, −0.40]	< 0.0001	95	< 0.00001		
Way of Intervention								
Face-to-face	27	1609/1624	−0.62 [−0.90, −0.34]	< 0.0001	93	< 0.00001	0.6	0.44
Telehealth	6	672/665	−0.78 [−1.07, −0.49]	< 0.00001	83	< 0.0001		
Intervention Category								
CBT	17	1367/1352	−0.45 [−0.64, −0.26]	< 0.00001	82	< 0.00001	4.37	0.36
IPT	11	720/738	−1.07 [−1.70, −0.44]	0.0008	96	< 0.00001		
MBI	3	89/96	−0.44 [−0.74, −0.15]	0.003	0	0.42		
BA	1	70/68	−0.33 [−0.67, 0.00]	0.02	NA	NA		
PST	1	35/35	−0.56 [−1.04, −0.08]	0.05	NA	NA		

### Meta-regression

Multiple meta-regressions identified three covariates significantly linked to effect size, indicating potential sources of heterogeneity: mean age (β = 0.062, 95% CI 0.007 to 0.117, *P* = 0.027), type of intervention (β = −0.674, 95% CI − 1.224 to − 0.124, *P* = 0.019), and intervention duration (β = 0.041, 95% CI 0.004 to 0.078, *P* = 0.031). Specifically, higher mean participant age and longer intervention duration were linked to larger effect sizes, while the specified intervention type showed smaller effects compared to the reference group (negative β). The other covariates in the multivariable model were not statistically significant (*P* > 0.05). Full regression coefficients are provided in the Supplementary Materials (Appendix S7).

## Disscussion

This systematic review and meta-analysis included 33 randomized controlled trials that examined the effectiveness of various psychological treatments for postpartum depression. Overall, psychotherapy significantly reduced postpartum depressive symptoms, with a moderate effect size, consistent with previous meta-analyses [8–54]. Notably, individualized therapy showed a higher effect size than group interventions in perinatal women, while interventions delivered by trained non-professional therapists appeared to have effect sizes similar to those of professional mental health providers. Additionally, interventions conducted in non-clinical settings (such as communities or homes) seemed more effective than those in clinical settings. Among psychological treatment options for perinatal depression, IPT had the highest effect size, followed by CBT, MBT, and BA.

### Study characteristics

Firstly, the average age of study participants may significantly influence treatment outcomes in psychological interventions for perinatal depression. In this study, younger pregnant women and new mothers, especially those aged 20 to 30, exhibited lower depression scores after psychological treatments. This improvement might be due to their greater physiological and psychological resilience, stronger social support networks, higher levels of treatment acceptance and adherence, and increased cognitive flexibility and emotional responsiveness [55–57]. These factors work together to produce more noticeable therapeutic effects. Future research should aim to develop strategies that maximize treatment results across different age groups of perinatal women.

Secondly, the greater reduction in depression scores seen in interventions lasting more than 8 weeks may result from the longer duration, which allows for the development of a strong therapeutic relationship between mothers and therapists. This extended period enables deeper exploration and resolution of underlying issues, helping patients establish and strengthen new behavioral and cognitive patterns. Additionally, longer interventions offer more chances to practice and master coping skills, build therapeutic benefits, and receive ongoing support — all of which aid patients in better adapting to and managing the challenges of the perinatal period. Furthermore, an extended treatment duration accommodates individual differences, helps prevent relapse, and ultimately leads to more lasting therapeutic results [2, 58].

Third, although the Edinburgh Postnatal Depression Scale (EPDS) is recognized as a standard screening tool for perinatal depression, there appears to be no significant difference in the effectiveness of psychological interventions for participants diagnosed with depressive symptoms using the EPDS compared to those diagnosed with other screening tools [59]. This indicates that cultural adaptations of screening tools are equally important when addressing perinatal depression among women from diverse cultural backgrounds.

Fourth, there is no significant difference in the effectiveness of psychological interventions in reducing depression scores between high-income countries and low- to middle-income countries. Previous research has shown that more than a quarter of perinatal women in low- to middle-income countries may be affected by depression [60]. Therefore, it is equally important to implement psychological interventions for pregnant women in these countries [61]. Future studies should explore the universal effectiveness of psychological interventions across different cultural and economic settings. Additionally, policies should aim to provide low- to middle-income countries with mental health resources and services comparable to those available in high-income countries, thereby promoting equal treatment outcomes.

### Components of the intervention programs

First, our study demonstrates that individualized treatment approaches produce better outcomes than group-based psychological interventions, with the former showing a higher effect size and the latter a lower one. This finding aligns with previous reviews [8, 62, 63]. This may be because individualized treatment can tailor interventions to a patient’s specific symptoms, medical history, and personal needs, thereby improving the precision and effectiveness of the therapy. The private setting allows mothers to express their emotions and concerns more freely, reducing stigma and providing deeper emotional support and psychological understanding [64]. Moreover, this environment alleviates the social pressures often linked to group settings, making patients feel more comfortable and relaxed. Individualized treatment also fosters trust and collaboration between the therapist and the patient, enhancing therapeutic outcomes. It allows for flexible treatment strategies that can be promptly adjusted based on the patient’s responses and progress, ensuring the adaptability of the process [65]. Therefore, our findings highlight the importance of personalized treatment, which typically requires a longer duration to provide sustained support and interventions, enabling women to better manage and cope with their symptoms.

Second, our analysis shows that psychological interventions have moderate effect sizes both during pregnancy and within the first year postpartum. This may be due to the significant physiological and psychological changes women experience during these times, including hormonal fluctuations, bodily changes, decreased sleep quality, role transitions, lifestyle adjustments, and social support variations [66–68]. These changes can lead to similar psychological issues, such as anxiety, depression, and emotional instability. Psychological therapy effectively addresses these common concerns, resulting in comparable outcomes during pregnancy and postpartum. Additionally, therapies like CBT and IPT have proven effective in treating anxiety and depression by identifying and changing negative thought patterns, strengthening coping skills, and improving emotional regulation [69–71]. Therefore, psychological therapy offers consistent support and coping strategies across both stages. As a result, research should aim to develop and implement more standardized intervention protocols to enhance the efficiency and effectiveness of these treatments.

Third, psychological treatments provided in non-medical settings show a high effect size, while those done in medical settings show a moderate effect size. Research indicates that patient engagement is positively linked to treatment outcomes. In non-medical settings, therapy often takes place in a more comfortable and informal environment, which helps patients relax, lowers psychological defenses, and encourages more open participation in therapy. The natural environment also boosts treatment effectiveness [72]. Additionally, non-medical settings give therapists more time and flexibility to build a strong therapeutic relationship with patients [73]. Studies have found that a solid therapeutic alliance is a key factor in successful psychotherapy and can greatly improve treatment outcomes [74, 75]. Lastly, non-medical settings enable therapists to provide a higher level of personalized treatment tailored to each patient’s needs. This personalized approach can better address specific issues and improve overall treatment effectiveness.

Fourth, the analysis shows that both professional psychotherapists and trained non-specialist providers (NSPs) have a moderate effect size in reducing perinatal depression scores. However, we notice that the current availability of professional psychotherapists is limited due to high costs, insufficient funding, lack of insurance coverage, and limited access [76]. In real-world settings, most patients in low- and middle-income countries seek treatment through primary healthcare or community-based platforms, where psychotherapy is often provided by NSPs through task-sharing [77]. The most common NSPs include community health workers employed by the health system, as well as peers, trial staff recruited from the same community, nurses, and midwives. Additionally, NSPs often come from the patient’s community, enabling them to offer more immediate and everyday social support [78]. This support goes beyond psychotherapy and includes help with daily life and emotional support, which is especially important during the perinatal period. Such comprehensive social support has been shown to significantly improve perinatal depression symptoms [79]. Therefore, future policies should focus on training non-specialist providers to effectively deliver therapeutic interventions.

Fifth, both telehealth and face-to-face therapy show moderate effect sizes in their outcomes, consistent with previous research findings [80–82]. The COVID-19 pandemic has greatly sped up the adoption and development of telehealth, leading to a significant rise in studies and digital interventions, especially those involving telehealth [83]. Despite this growth, most digital interventions still focus on cognitive behavioral therapy (CBT), either as self-guided modules or as asynchronously guided by therapists [84, 85]. Recent studies indicate that telehealth is a practical alternative for managing residual depressive symptoms in perinatal women and offers vital support to those in remote areas [86–88]. However, other research recommends that future studies perform individual patient data meta-analyses to improve and personalize telehealth interventions [89].

Finally, our study shows that IPT has a high effect size in treating perinatal depression, while CBT, MBI, and BA exhibit lower effect sizes [62]. PST did not show significant effectiveness for perinatal depression. More research is needed to confirm the effects of MBI, BA, and PST [90]. Our findings align with previous studies, and we offer the following explanations: earlier research has identified ongoing interpersonal issues as a key risk factor for perinatal depression, highlighting the importance of personalized interventions that target these issues [54]. IPT, which emphasizes enhancing social support, improving interpersonal relationships, and managing role transitions, is especially effective in addressing interpersonal challenges. This therapy helps women cope with the unique stressors during the perinatal period, promotes emotional expression and problem-solving [91], reduces psychological burden, and bolsters coping mechanisms.

But recommending IPT as the preferred treatment requires a thorough assessment of feasibility, scalability, and practical comparisons. IPT is typically provided in time-limited, weekly sessions (usually 8–16 sessions), and like manualized CBT, relies on structured training, supervision, and quality monitoring [92]. Health systems looking to expand IPT should examine four areas: workforce and training, patient- and service-level time, infrastructure comparison with CBT, and delivery models that can broaden access [93, 94]. Regarding workforce and training, IPT competence generally depends on didactic learning, case-based practice, and ongoing supervision; in settings with few specialist IPT therapists, structured training for non-specialist providers with supervisory support (task-sharing) may be a practical option [78, 95]. Implementation plans should detail training components (such as an introductory workshop, a set number of supervised cases, and fidelity checks), supervision frequency, and the competency levels needed for independent practice. At the patient and service level, the resource use per session and over the course of treatment for IPT is comparable to many CBT protocols [79], but patient acceptance may be affected by treatment focus (interpersonal issues versus cognitive restructuring) and delivery mode (in-person versus remote) [78, 96, 97]. Group, brief-format, or remote IPT adaptations might reduce access barriers. A stepped-care approach—offering low-intensity interventions initially and escalating non-responders to IPT—could help extend reach within limited resources.

In pragmatic comparisons, CBT benefits from more established online curricula, training pathways, and reimbursement mechanisms in many systems, which facilitate rapid scale-up; by contrast, IPT implementation may require upfront investment in training, supervision, and digital materials [79, 98]. We therefore recommend implementation research—particularly hybrid effectiveness–implementation trials and economic evaluations—that directly compare IPT and CBT in routine services on effectiveness, cost-effectiveness, acceptability, reach, and equity to inform policy and clinical decision-making.However, some studies show results that conflict with ours [20]. This difference may be because our included studies did not restrict the severity of perinatal depression, while the referenced study focused solely on severe cases. Based on our findings, we recommend IPT as the primary treatment for effectively managing perinatal depression. However, due to the unique characteristics of the perinatal depression population and the lack of direct comparisons among these therapies, future research should conduct network meta-analyses to determine the most effective treatments for perinatal depression at various stages and severities.

### Strengths and limitations

In this review, we conducted a comprehensive search and included a large sample of studies on perinatal depression. The current systematic review and meta-analysis focus exclusively on RCTs to help depressed mothers make informed choices and clinical decisions based on the highest-quality evidence. Few existing reviews address specific intervention aspects, such as timing (during pregnancy and postpartum), setting, target populations, and implementation methods. We included existing studies in various subgroup analyses. Additionally, our findings indicate that personalized psychological interventions in non-medical settings may improve treatment outcomes for mothers with perinatal depression. Therefore, our assessment of current evidence on psychological interventions offers clinically relevant guidance for pregnant and new mothers, as well as for clinicians and researchers.

This study has several limitations. We excluded trials that involved additional pharmacotherapy during the perinatal period, and some intervention subgroups (for example, MBT, BA, PST) include relatively few studies. Only English-language publications were considered, and trials published in other languages or as grey literature may have been omitted. There was significant variability in treatment dose and duration across trials, with session lengths ranging from 30 min to 2 h, which makes identifying an optimal regimen difficult.

We examined heterogeneity through subgroup analysis, sensitivity analysis, and multivariate random-effects meta-regression. However, besides the causes identified by meta-regression, there may be unmeasured study-level moderating factors such as disease severity, comorbidities, therapist adherence or experience, and participant adherence. Notably, some older studies did not provide sufficient reports on trial dropout rates and participant adherence, preventing formal meta-analyses. The incomplete reporting of these measures is a key limitation. Future trials should report standardized compliance and dropout rates and consider coordinating intervention doses and outcome assessments to improve comparability. Additionally, study quality is another concern: out of 33 trials, only 11 were rated as having a low risk of bias, and widespread issues included participants being unable to be blinded and implementation bias. Such biases may lead to an overestimation of effects. In behavioral intervention trials where blinding isn’t feasible, the expected effects might appear more favorable for the intervention group. When outcomes are self-reported (e.g., depression scales), the inability to blind outcome assessors could introduce detection bias. Systematic differences in adherence between groups could also result in performance bias affecting effect estimates.

## Conclusion

In summary, this systematic review and meta-analysis of psychological interventions for perinatal depression shows that interpersonal psychotherapy (IPT), cognitive behavioral therapy (CBT), mindfulness-based intervention (MBI), and biofeedback therapy (BA) are all effective in reducing perinatal depression. Meanwhile, trauma-focused therapy (PST) may be less effective, though more research is needed for a clearer evaluation. Additionally, IPT has a higher effect size compared to other psychotherapies. This study also highlights the importance of study design and components in improving psychotherapy outcomes, especially in personalized treatments conducted outside medical settings. Furthermore, trained non-professional psychotherapists can also produce significant therapeutic results in psychological interventions.

## Supplementary Information

Supplementary Material 1.

## Data Availability

The authors confirm that the data supporting the findings of this study are available within the study and the Supplemental Material. Raw data supporting the study’s findings are available from the corresponding author upon reasonable request.
