# Supplementary material for: Different psychological interventions for perinatal depression: a systematic review and meta-analysis of randomized controlled trials
Source: BMC Psychiatry. 2025 Oct 14;25:981. doi: 10.1186/s12888-025-07462-3 (PMC12522513; doi:10.1186/s12888-025-07462-3)
Supplement: Supplementary file 1 — Supplementary Material 1. [file 12888_2025_7462_MOESM1_ESM.docx]

**Supplementary appendix**

**Different Psychological Interventions for Perinatal Depression: A Systematic Review and Meta-Analysis of Randomized Controlled Trials**

Guangshun Hua, Keling Yue, Yu Zhu, Fuchao Yang, Mijuan Zhou

**Online Supporting Material**

Appendix S1. PRISMA Guidelines Checklist

| **Section and Topic** | **Item #** | **Checklist item** | **Location where item is reported** |
| --- | --- | --- | --- |
| **TITLE** | | |  |
| Title | 1 | Identify the report as a systematic review. | **1** |
| **ABSTRACT** | | |  |
| Abstract | 2 | See the PRISMA 2020 for Abstracts checklist. | **1** |
| **INTRODUCTION** | | |  |
| Rationale | 3 | Describe the rationale for the review in the context of existing knowledge. | **2** |
| Objectives | 4 | Provide an explicit statement of the objective(s) or question(s) the review addresses. | **2-3** |
| **METHODS** | | |  |
| Eligibility criteria | 5 | Specify the inclusion and exclusion criteria for the review and how studies were grouped for the syntheses. | **3** |
| Information sources | 6 | Specify all databases, registers, websites, organisations, reference lists, and other sources searched or consulted to identify studies. Specify the date when each source was last searched or consulted. | **3** |
| Search strategy | 7 | Present the full search strategies for all databases, registers, and websites, including any filters and limits used. | **3-4** |
| Selection process | 8 | Specify the methods used to decide whether a study met the inclusion criteria of the review, including how many reviewers screened each record and each report retrieved, whether they worked independently, and, if applicable, details of automation tools used in the process. | **3-4** |
| Data collection process | 9 | Specify the methods used to collect data from reports, including how many reviewers collected data from each report, whether they worked independently, any processes for obtaining or confirming data from study investigators, and, if applicable, details of automation tools used in the process. | **3-4** |
| Data items | 10a | List and define all outcomes for which data were sought. Specify whether all results that were compatible with each outcome domain in each study were sought (e.g., for all measures, time points, analyses), and if not, the methods used to decide which results to collect. | **3-4** |
|  | 10b | List and define all other variables for which data were sought (e.g., participant and intervention characteristics, funding sources). Describe any assumptions made about any missing or unclear information. | **3-4** |
| Study risk of bias assessment | 11 | Specify the methods used to assess risk of bias in the included studies, including details of the tool(s) used, how many reviewers assessed each study, and whether they worked independently, and if applicable, details of automation tools used in the process. | **4** |
| Effect measures | 12 | Specify for each outcome the effect measure(s) (e.g., risk ratio, mean difference) used in the synthesis or presentation of results. | **4** |
| Synthesis methods | 13a | Describe the processes used to decide which studies were eligible for each synthesis (e.g. tabulating the study intervention characteristics and comparing against the planned groups for each synthesis (item #5)). | **4** |
|  | 13b | Describe any methods required to prepare the data for presentation or synthesis, such as handling of missing summary statistics or data conversions. | **4** |
|  | 13c | Describe any methods used to tabulate or visually display results of individual studies and syntheses. | **4** |
|  | 13d | Describe any methods used to synthesize results and provide a rationale for the choice(s). If meta-analysis was performed, describe the model(s), method(s) to identify the presence and extent of statistical heterogeneity, and software package(s) used. | **4** |
|  | 13e | Describe any methods used to explore possible causes of heterogeneity among study results (e.g., subgroup analysis, meta-regression). | **4** |
|  | 13f | Describe any sensitivity analyses conducted to assess robustness of the synthesized results. | **4** |
| Reporting bias assessment | 14 | Describe any methods used to assess the risk of bias due to missing results in a synthesis (arising from reporting biases). | **4** |
| Certainty assessment | 15 | Describe any methods used to assess certainty (or confidence) in the body of evidence for an outcome. | **4** |
| **RESULTS** | | |  |
| Study selection | 16a | Describe the results of the search and selection process, from the number of records identified in the search to the number of studies included in the review, ideally using a flow diagram. | **4-13** |
|  | 16b | Cite studies that might appear to meet the inclusion criteria, but which were excluded, and explain why they were excluded. | **4-13** |
| Study characteristics | 17 | Cite each included study and present its characteristics. | **4-13** |
| Risk of bias in studies | 18 | Present assessments of risk of bias for each included study. | **4-13** |
| Results of individual studies | 19 | For all outcomes, present, for each study: (a) summary statistics for each group (where appropriate) and (b) an effect estimate and its precision (e.g. confidence/credible interval), ideally using structured tables or plots. | **4-13** |
| Results of syntheses | 20a | For each synthesis, briefly summarise the characteristics and risk of bias among contributing studies. | **4-13** |
|  | 20b | Present the results of all statistical syntheses conducted. If meta-analysis was done, present for each the summary estimate and its precision (e.g., confidence/credible interval) and measures of statistical heterogeneity. If comparing groups, describe the direction of the effect. | **4-13** |
|  | 20c | Present results of all investigations of possible causes of heterogeneity among study results. | **4-13** |
|  | 20d | Present the results of all sensitivity analyses conducted to assess the robustness of the synthesized results. | **4-13** |
| Reporting biases | 21 | Present assessments of risk of bias due to missing results (arising from reporting biases) for each synthesis assessed. | **4-13** |
| Certainty of evidence | 22 | Present assessments of certainty (or confidence) in the body of evidence for each outcome assessed. | **4-13** |
| **DISCUSSION** | | |  |
| Discussion | 23a | Provide a general interpretation of the results in the context of other evidence. | **13-17** |
|  | 23b | Discuss any limitations of the evidence included in the review. | **13-17** |
|  | 23c | Discuss any limitations of the review processes used. | **13-17** |
|  | 23d | Discuss implications of the results for practice, policy, and future research. | **13-17** |
| **OTHER INFORMATION** | | |  |
| Registration and protocol | 24a | Provide registration information for the review, including register name and registration number, or state that the review was not registered. | **3** |
|  | 24b | Indicate where the review protocol can be accessed, or state that a protocol was not prepared. | **18** |
|  | 24c | Describe and explain any amendments to information provided at registration or in the protocol. | **18** |
| Support | 25 | Describe sources of financial or non-financial support for the review, and the role of the funders or sponsors in the review. | **18** |
| Competing interests | 26 | Declare any competing interests of review authors. | **18** |
| Availability of data, code, and other materials | 27 | Report which of the following are publicly available and where they can be found: template data collection forms; data extracted from included studies; data used for all analyses; analytic code; any other materials used in the review. | **18** |

Appendix S2. Search strategy

**S2.1 EBM Reviews - Cochrane Central Register of Controlled Trials <February 2024>**

1 exp Depression/ 17901

2 "depress*".mp. 114226

3 exp Dysthymic Disorder/ 200

4 "dysthymi*".mp. 1049

5 exp Mood Disorders/ 17441

6 ("mood disorder*" or "affective disorder*").mp. 5884

7 or/1-6 116829

8 exp Pregnancy/ 33135

9 "Pregnancy".ti, ab. 53919

10 exp Parity/ 1186

11 ("Primiparity" or "Multiparity").ti,ab. 148

12 exp pregnancy trimesters/ 2334

13 "pregnancy trimesters".ti, ab. 8

14 exp pregnancy trimester, first/ or exp pregnancy trimester, second/ or exp pregnancy trimester, third/ 2253

15 exp Pregnant Women/ 982

16 exp Postpartum Period/ 2724

17 ("Postpartum Period" or "Postpartum" or "Puerperium").ti, ab. 12832

18 exp perinatal care/ 824

19 "perinatal care".ti, ab. 186

20 exp postnatal care/ 575

21 "postnatal care".ti,ab. 406

22 exp Prenatal Care/ 2281

23 ("Prenatal care" or "Antenatal care").ti,ab. 2945

24 or/8-23 78000

25 exp Psychotherapy/ 34728

26 "Psychotherap*".mp. 17278

27 "psychological".mp. 66520

28 "art therapy".mp. 596

29 "assertive training".mp. 70

30 "autogenic training".mp. 342

31 "aversion therapy".mp. 33

32 "Balint group".mp. 19

33 "behavior contracting".mp. 16

34 "behavior modification".mp. 1016

35 "behavior therapy".mp. 11086

36 "dialectical behavior therapy".mp. 431

37 "sleep phase chronotherapy".mp. 6

38 "bibliotherapy".mp. 341

39 "biofeedback therapy".mp. 311

40 "Color Therapy".mp. 34

41 "Crisis Intervention".mp. 527

42 "body psychotherapy".mp. 36

43 "catharsis".mp. 84

44 "client centered therapy".mp. 61

45 "cognitive rehabilitation".mp. 1669

46 "cognitive therapy".mp. 7089

47 ("cognitive behavioral therapy" or "CBT").mp. 21814

48 "cognitive remediation".mp. 1238

49 "couple therapy".mp. 228

50 "dance therapy".mp. 254

51 "drama therapy".mp. 25

52 "emotion regulation training".mp. 111

53 "emotion-focused therapy".mp. 109

54 "family therapy".mp. 1933

55 "interpersonal psychotherapy".mp. 727

56 "Gestalt therapy".mp. 35

57 "Autogenic Training".mp. 342

58 "group therapy".mp. 3872

59 "guided imagery".mp. 908

60 "interpersonal psychotherapy".mp. 727

61 "Logotherapy".mp. 59

62 "mentalization-based treatment".mp. 78

63 "music therapy".mp. 3115

64 "narrative therapy".mp. 140

65 "Person-Centered Psychotherapy".mp. 94

66 "play therapy*".mp. 320

67 "psychoanalytic therapy".mp. 198

68 "psychodynamic psychotherapy".mp. 335

69 "psychodynamic therapy*".mp. 170

70 "psychosocial intervention".mp. 1836

71 "rational emotive behavior therapy".mp. 72

72 "reality therapy".mp. 202

73 "relaxation training".mp. 2154

74 ("metacognition therap*" or "meta cognition therap*").mp. 4

75 "role play".mp. 570

76 "schema therapy".mp. 186

77 ("self control*" or "self talk" or "self help").mp. 7489

78 "sociotherapy".mp. 19

79 "social skill".mp. 159

80 "socioenvironmental therapy".mp. 49

81 "solution-focused therapy".mp. 91

82 "telepsychotherapy".mp. 58

83 "therapeutic community".mp. 150

84 "supportive therapy".mp. 997

85 "physical reinforcement therapy".mp. 0

86 "exposure therapy*".mp. 1801

87 "consultation therap*".mp. 14

88 "client-centered ".mp. 288

89 "problem solving".mp. 6822

90 or/25-89 125741

91 exp Depression, Postpartum/ 964

92 ("Depression, Postpartum" or "Postpartum depression").mp. 1784

93 exp Puerperal Disorders/ 2550

94 "Puerperal Disorder*".mp. 382

95 "perinatal depression".mp. 345

96 "antenatal depression".mp. 177

97 "postnatal depression".mp. 1738

98 "baby blue".mp. 1

99 or/91-98 4480

100 exp Randomized Controlled Trial/ 37

101 "Randomized Controlled Trial".ti,ab. 176846

102 "randomized".ti,ab. 856646

103 "randomised".ti,ab. 168737

104 "randomly".ti, ab. 324165

105 "placebo".ti,ab. 366683

106 exp Clinical Trials as Topic/ 89329

107 "double-blind".ti,ab. 263324

108 "trial".ti,ab. 787710

109 or/100-108 1442058

110 7 and 24 and 90 and 109 1387

111 90 and 99 and 109 967

112 110 or 111 1606

**S2.2 Embase <1974 to 2024 March 11>**

1 exp depression/ 645676

2 "depress*".mp. 1006366

3 exp Dysthymic Disorder/ 11952

4 "dysthymi*".mp. 10691

5 exp mood disorder/ 696139

6 ("mood disorder*" or "affective disorder*").mp. 92825

7 or/1-6 1100597

8 exp pregnancy/ 793327

9 "Pregnancy".ti, ab. 604006

10 exp Parity/ 45873

11 ("Primiparity" or "Multiparity").ti,ab. 4004

12 exp pregnancy trimesters/ 793327

13 "pregnancy trimesters".ti,ab. 272

14 exp pregnancy trimester, first/ or exp pregnancy trimester, second/ or exp pregnancy trimester, third/ 99233

15 exp Pregnant Women/ 118378

16 exp Postpartum Period/ 84071

17 ("Postpartum Period" or "Postpartum" or "Puerperium").ti, ab. 97743

18 exp perinatal care/ 72853

19 "perinatal care".ti, ab. 3986

20 exp postnatal care/ 140379

21 "postnatal care".ti,ab. 3146

22 exp Prenatal Care/ 183888

23 ("Prenatal care" or "Antenatal care").ti,ab. 31088

24 or/8-23 1246166

25 exp psychotherapy/ 304113

26 "Psychotherap*".mp. 128555

27 "psychological".mp. 897878

28 "art therapy".mp. 5666

29 "assertive training".mp. 409

30 "autogenic training".mp. 1411

31 "aversion therapy".mp. 647

32 "Balint group".mp. 535

33 "behavior contracting".mp. 69

34 "behavior modification".mp. 10771

35 "behavior therapy".mp. 52998

36 "dialectical behavior therapy".mp. 1853

37 "sleep phase chronotherapy".mp. 3

38 "bibliotherapy".mp. 775

39 "biofeedback therapy".mp. 1199

40 "Color Therapy".mp. 171

41 "Crisis Intervention".mp. 7672

42 "body psychotherapy".mp. 177

43 "catharsis".mp. 624

44 "client centered therapy".mp. 516

45 "cognitive rehabilitation".mp. 6148

46 "cognitive therapy".mp. 46509

47 ("cognitive behavioral therapy" or "CBT").mp. 47791

48 "cognitive remediation".mp. 3113

49 "couple therapy".mp. 1452

50 "dance therapy".mp. 982

51 "drama therapy".mp. 235

52 "emotion regulation training".mp. 122

53 "emotion-focused therapy".mp. 362

54 "family therapy".mp. 15386

55 "interpersonal psychotherapy".mp. 1637

56 "gestalt therapy".mp. 1108

57 "Autogenic Training".mp. 1411

58 "group therapy".mp. 23063

59 "guided imagery".mp. 2420

60 "interpersonal psychotherapy".mp. 1637

61 "Logotherapy".mp. 213

62 "mentalization-based treatment".mp. 408

63 "music therapy".mp. 9950

64 "narrative therapy".mp. 741

65 "Person-Centered Psychotherapy".mp. 13

66 "play therapy*".mp. 2103

67 "psychoanalytic therapy".mp. 444

68 "psychodynamic psychotherapy".mp. 2978

69 "psychodynamic therapy*".mp. 1124

70 "psychosocial intervention".mp. 5822

71 "rational emotive behavior therapy".mp. 340

72 "reality therapy".mp. 423

73 "relaxation training".mp. 13241

74 ("metacognition therap*" or "meta cognition therap*").mp. 6

75 "role play".mp. 3115

76 "schema therapy".mp. 535

77 ("self control*" or "self talk" or "self help").mp. 40643

78 "sociotherapy".mp. 585

79 "social skill".mp. 1099

80 "socioenvironmental therapy".mp. 1

81 "solution-focused therapy".mp. 235

82 "telepsychotherapy".mp. 447

83 "therapeutic community".mp. 3148

84 "supportive therapy".mp. 7635

85 "physical reinforcement therapy".mp. 0

86 "exposure therapy*".mp. 4786

87 "consultation therap*".mp. 45

88 "client-centered ".mp. 1954

89 "problem solving".mp. 52157

90 or/25-89 1236074

91 exp postnatal depression/ 7912

92 "postnatal depression".mp. 13721

93 ("Depression, Postpartum" or "Postpartum depression").mp. 8229

94 exp perinatal depression/ 9880

95 "perinatal depression".mp. 2720

96 exp puerperal disorder/ 59844

97 "Puerperal Disorder*".mp. 3963

98 exp antenatal depression/ 1312

99 "antenatal depression".mp. 1894

100 "baby blue".mp. 19

101 or/91-100 70370

102 exp randomized controlled trial/ 813401

103 "randomized controlled trial".ti, ab. 144419

104 "randomized".ti, ab. 999192

105 "randomised".ti,ab. 198916

106 "randomly".ti, ab. 571507

107 "placebo".ti,ab. 373951

108 exp "clinical trial (topic)"/ 457551

109 "double-blind".ti,ab. 227673

110 "trial".ti,ab. 1159771

111 or/102-110 2647033

112 7 and 24 and 90 and 111 1803

113 90 and 101 and 111 1320

114 112 or 113 2154

**S2.3 Ovid MEDLINE(R) and Epub Ahead of Print, In-Process, In-Data-Review & Other Non-Indexed Citations, Daily and Versions <1946 to March 11, 2024>**

1 exp Depression/ 156053

2 "depress*".mp. 672174

3 exp Dysthymic Disorder/ 1178

4 "dysthymi*".mp. 3819

5 exp Mood Disorder/ 172819

6 ("mood disorder*" or "affective disorder*").mp. 51826

7 or/1-6 717324

8 exp pregnancy/ 1026036

9 "Pregnancy".ti, ab. 462902

10 exp Parity/ 26859

11 ("Primiparity" or "Multiparity").ti,ab. 2934

12 exp pregnancy trimesters/ 45323

13 "pregnancy trimesters".ti, ab. 194

14 exp pregnancy trimester, first/ or exp pregnancy trimester, second/ or exp pregnancy trimester, third/ 42975

15 exp Pregnant Women/ 15648

16 exp Postpartum Period/ 77634

17 ("Postpartum Period" or "Postpartum" or "Puerperium").ti, ab. 76158

18 exp perinatal care/ 11742

19 "perinatal care".ti, ab. 3137

20 exp postnatal care/ 6494

21 "postnatal care".ti,ab. 2747

22 exp Prenatal Care/ 33210

23 ("Prenatal care" or "Antenatal care").ti,ab. 25316

24 or/8-23 1170297

25 exp Psychotherapy/ 222395

26 "Psychotherap*".mp. 102316

27 "psychological".mp. 686709

28 "art therapy".mp. 2522

29 "assertive training".mp. 59

30 "autogenic training".mp. 1351

31 "aversion therapy".mp. 185

32 "Balint group".mp. 187

33 "behavior contracting".mp. 11

34 "behavior modification".mp. 2519

35 "behavior therapy".mp. 35378

36 "dialectical behavior therapy".mp. 934

37 "sleep phase chronotherapy".mp. 18

38 "bibliotherapy".mp. 656

39 "biofeedback therapy".mp. 680

40 "Color Therapy".mp. 108

41 "Crisis Intervention".mp. 7178

42 "body psychotherapy".mp. 37

43 "catharsis".mp. 555

44 "client-centered therapy".mp. 91

45 "cognitive rehabilitation".mp. 2457

46 "cognitive therapy".mp. 3911

47 ("cognitive behavioral therapy" or "CBT").mp. 41735

48 "cognitive remediation".mp. 1796

49 "couple therapy".mp. 572

50 "dance therapy".mp. 565

51 "drama therapy".mp. 83

52 "emotion regulation training".mp. 83

53 "emotion-focused therapy".mp. 187

54 "family therapy".mp. 10457

55 "interpersonal psychotherapy".mp. 1076

56 "Gestalt Therapy".mp. 200

57 "Autogenic Training".mp. 1351

58 "group therapy".mp. 5214

59 "guided imagery".mp. 927

60 "interpersonal psychotherapy".mp. 1076

61 "Logotherapy".mp. 150

62 "mentalization-based treatment".mp. 176

63 "music therapy".mp. 5827

64 "narrative therapy".mp. 431

65 "Person-Centered Psychotherapy".mp. 458

66 "play therap*".mp. 1458

67 "psychoanalytic therapy".mp. 15493

68 "psychodynamic psychotherapy".mp. 1185

69 "psychodynamic therapy*".mp. 727

70 "psychosocial intervention".mp. 3723

71 "rational emotive behavior therapy".mp. 104

72 "reality therapy".mp. 498

73 "relaxation training".mp. 1467

74 ("metacognition therap*" or "meta cognition therap*").mp. 4

75 "role play".mp. 1965

76 "schema therapy".mp. 334

77 ("self control*" or "self talk" or "self help").mp. 34819

78 "sociotherapy".mp. 154

79 "social skill".mp. 730

80 "socioenvironmental therapy".mp. 463

81 "solution-focused therapy".mp. 98

82 "telepsychotherapy".mp. 64

83 "therapeutic community".mp. 2629

84 "supportive therapy".mp. 4850

85 "physical reinforcement therapy".mp. 0

86 "exposure therap*".mp. 3403

87 "consultation therap*".mp. 29

88 "client centered".mp. 1217

89 "problem solving".mp. 42867

90 or/25-89 951612

91 exp Depression, Postpartum/ 7773

92 ("Depression, Postpartum" or "Postpartum depression").mp. 9988

93 exp Puerperal Disorders/ 38873

94 "Puerperal Disorder*".mp. 11830

95 "perinatal depression".mp. 1730

96 "antenatal depression".mp. 863

97 "postnatal depression".mp. 5079

98 "baby blue".mp. 9

99 or/91-98 43764

100 exp Randomized Controlled Trial/ 611496

101 "randomized controlled trial".pt. 609904

102 "controlled clinical trial".pt. 95581

103 "pragmatic clinical trial".pt. 2332

104 "randomized".ti, ab. 694456

105 "randomised".ti,ab. 136590

106 "randomly".ti, ab. 429870

107 "placebo".ti,ab. 253151

108 exp Clinical Trials as Topic/ 388777

109 "double-blind".ti,ab. 159743

110 "trial".ti,ab. 797268

111 or/100-110 1951424

112 7 and 24 and 90 and 111 1242

113 90 and 99 and 111 826

114 112 or 113 1403

**S2.4 APA PsycInfo <1806 to March Week 2 2024>**

1 exp "Depression (Emotion)"/ 27175

2 "depress*".ti,ab. 362158

3 exp Dysthymic Disorder/ 1531

4 "dysthymi*".ti,ab. 3869

5 exp Mood Disorders/ 181032

6 ("mood disorder*" or "affective disorder*").ti,ab. 34944

7 or/1-6 390646

8 exp Pregnancy/ 50698

9 "Pregnancy".ti, ab. 43821

10 ("Primiparity" or "Multiparity").ti,ab. 228

11 "pregnancy trimesters".ti, ab. 38

12 exp Postnatal Period/ 7020

13 "Postnatal Period".ti,ab. 1464

14 ("Postpartum Period" or "Postpartum" or "Puerperium").ti, ab. 15164

15 exp Prenatal Care/ 2614

16 ("Prenatal care" or "Antenatal care").ti,ab. 3752

17 ("postnatal care" or "perinatal care").ti, ab. 717

18 or/8-17 79938

19 exp Psychotherapy/ 225726

20 "Psychotherap*".mp. 218452

21 "psychological".mp. 596135

22 exp Supportive Psychotherapy/ or exp Psychodynamic Psychotherapy/ 4714

23 "art therapy".mp. 6899

24 "assertive training".mp. 309

25 "autogenic training".mp. 1067

26 "aversion therapy".mp. 749

27 "Balint group".mp. 190

28 "behavior contracting".mp. 341

29 "behavior modification".mp. 15737

30 "behavior therapy".mp. 59791

31 "dialectical behavior therapy".mp. 2705

32 "sleep phase chronotherapy".mp. 9

33 "bibliotherapy".mp. 1477

34 "biofeedback therapy".mp. 173

35 "Color Therapy".mp. 26

36 "Crisis Intervention".mp. 9317

37 "body psychotherapy".mp. 343

38 "catharsis".mp. 1667

39 "client centered therapy".mp. 3941

40 "cognitive rehabilitation".mp. 4056

41 "cognitive therapy".mp. 27227

42 ("cognitive behavioral therapy" or "CBT").mp. 29198

43 "cognitive remediation".mp. 1798

44 "couple therapy".mp. 2752

45 "dance therapy".mp. 1627

46 "drama therapy".mp. 561

47 "emotion regulation training".mp. 98

48 "emotion-focused therapy".mp. 1164

49 "family therapy".mp. 30411

50 "interpersonal psychotherapy".mp. 2326

51 "gestalt therapy".mp. 2474

52 "Autogenic Training".mp. 1067

53 "group therapy".mp. 14826

54 "guided imagery".mp. 1710

55 "interpersonal psychotherapy".mp. 2326

56 "Logotherapy".mp. 942

57 "mentalization-based treatment".mp. 357

58 "music therapy".mp. 7022

59 "narrative therapy".mp. 2004

60 "Person-Centered Psychotherapy".mp. 120

61 "play therap*".mp. 5675

62 "psychoanalytic therapy".mp. 11035

63 "psychodynamic psychotherapy".mp. 5396

64 "psychodynamic therap*".mp. 2181

65 "psychosocial intervention".mp. 2839

66 "rational emotive behavior therapy".mp. 2164

67 "reality therap*".mp. 1290

68 "relaxation training".mp. 2747

69 "relaxation therap*".mp. 5496

70 ("metacognition therap*" or "meta cognition therap*").mp. 5

71 exp Metacognition/ or exp Metacognitive Therapy/ 9940

72 ("Metacognition" or "Metacognitive Therapy").mp. 12158

73 "role play".mp. 3290

74 "schema therapy".mp. 772

75 ("self control*" or "self talk" or "self help").mp. 36599

76 "sociotherapy".mp. 303

77 "social skill".mp. 2063

78 "socioenvironmental therapy".mp. 200

79 "solution-focused therapy".mp. 1223

80 "telepsychotherap*".mp. 87

81 "therapeutic community".mp. 4506

82 "supportive therapy".mp. 848

83 "physical reinforcement therap*".mp. 0

84 "exposure therap*".mp. 4908

85 "consultation therap*".mp. 50

86 "client centered".mp. 5448

87 "problem solving".mp. 68994

88 or/19-87 1028038

89 exp Postpartum Depression/ 6341

90 ("Postnatal Depression" or "Postnatal Dysphoria" or "Puerperal Depression").mp. 5786

91 "Puerperal Disorder*".mp. 973

92 "antenatal depression".mp. 513

93 "perinatal depression".mp. 1151

94 "baby blue".mp. 2

95 or/89-94 10393

96 exp Randomized Controlled Trials/ 1575

97 "randomized controlled trial".ti, ab. 24884

98 "controlled clinical trial".ti, ab. 1840

99 "pragmatic clinical trial".ti, ab. 53

100 "randomized".ti, ab. 96473

101 "randomised".ti,ab. 13782

102 "randomly".ti, ab. 86082

103 "placebo".ti,ab. 44316

104 clinical trials as topic/ 0

105 "trial".ti,ab. 129938

106 or/96-105 269376

107 7 and 18 and 88 and 106 514

108 88 and 95 and 106 486

109 107 or 108 664

Appendix S3. Risk of Bias Table for Studies, assessed via Cochrane Risk of Bias 2 Tool for Randomized Controlled Trials.

| **Author, Year, Country** | **Bias from randomization** | **Bias due to deviations from the intended intervention** | **Bias due to missing outcome data** | **Bias in the measurement of the outcome** | **Bias in the selection of the reported result** | **Overall Risk of Bias** |
| --- | --- | --- | --- | --- | --- | --- |
| Zoryana Babiy et al.,2024, Canada | Low | Some concerns | Low | Low | Low | Low |
| Donya Merza et al.,2023, Canada | Low | Low | Low | Low | Low | Low |
| Sanaa Abujilban et al.,2023, Jordanian | Some concerns | Low | Some concerns | Low | Low | Some concerns |
| Ryan J Van Lieshout et al.,2021,Canada | Low | Low | Low | Low | Low | Low |
| Cindy-Lee Dennis et al.,2020, Canada | Low | Some concerns | Low | Low | Low | Low |
| Erik Forsell et al.,2017,Sweden | Some concerns | Low | Low | Some concerns | Low | Some concerns |
| Susan Gennaro et al.,2024, United States | Some concerns | Some concerns | Low | Low | Low | Some concerns |
| Xuan Zhang et al.,2023, China | Some concerns | Low | Low | Some concerns | Low | Some concerns |
| Nusrat Husain et al.,2023, United Kingdom | Low | Low | Low | Low | Low | Low |
| Ryan J Van Lieshout et al.,2023,Canada | Low | Low | Low | Low | Low | Low |
| Benjamin L Hankin et al.,2023, United States | Low | Low | Low | Low | Low | Low |
| Ryan J Van Lieshout et al.,2022,Canada | Low | Low | Low | Low | Low | Low |
| Obadia Yator et al.,2022, Kenya | Some concerns | Some concerns | Low | Some concerns | Some concerns | Some concerns |
| Bahar Amani et al.,2022, Canada | Some concerns | High | High | High | Some concerns | High |
| Fei-Wan Ngai et al.,2022,China | Low | Low | Low | Low | Low | Low |
| Hongling Liu et al.,2021, China | High | High | High | Some concerns | Low | High |
| Feryal Khamseh et al.,2019, Iran | Some concerns | Low | Some concerns | Low | Some concerns | Some concerns |
| Shannon N Lenze et al.,2017, United States | Some concerns | Low | Low | Some concerns | Low | Some concerns |
| Sona Dimidjian et al.,2017, United States | Low | Some concerns | Low | Low | Low | Some concerns |
| S S K Leung et al.,2016, China | High | Some concerns | Some concerns | High | Some concerns | High |
| Sona Dimidjian et al.,2016, United States | High | High | High | High | Some concerns | High |
| Huaiyu Zhang et al.,2015, United States | Some concerns | Low | Low | Low | Low | Some concerns |
| Ali Fathi-Ashtiani et al.,2015, Iran | Some concerns | Low | Low | Some concerns | Low | Some concerns |
| Ling-ling Gao et al.,2015, China | Low | Low | Low | Low | Low | Low |
| Marla McGregor et al.,2014, Canada | Some concerns | Some concerns | Some concerns | Low | Low | Some concerns |
| Heather O'Mahen et al.,2013, United States | Low | Some concerns | Low | Low | Low | Some concerns |
| Alison Burns et al.,2013, United Kingdom | Some concerns | Low | Low | Some concerns | Some concerns | Some concerns |
| Huynh-Nhu Le et al.,2011, United States | Low | Some concerns | Low | Low | Low | Some concerns |
| Rhiannon Mulcahy et al.,2010, Australian | Low | Low | Low | Low | Low | Low |
| Nancy K Grote et al.,2009, United States | Some concerns | Low | High | Low | Low | High |
| C Zlotnick et al.,2001, United States | Some concerns | Low | Low | Low | Low | Some concerns |
| J. Prendergast et al.,2000, Australian | High | Some concerns | High | High | Some concerns | High |
| M. W. O'Hara et al.,2000, United States | Some concerns | Some concerns | Low | Low | Some concerns | Some concerns |

Appendix S4. The funnel plot and Egger's test were used to assess publication bias in the efficacy of psychological interventions for depression.

S4.1 The funnel plot

**
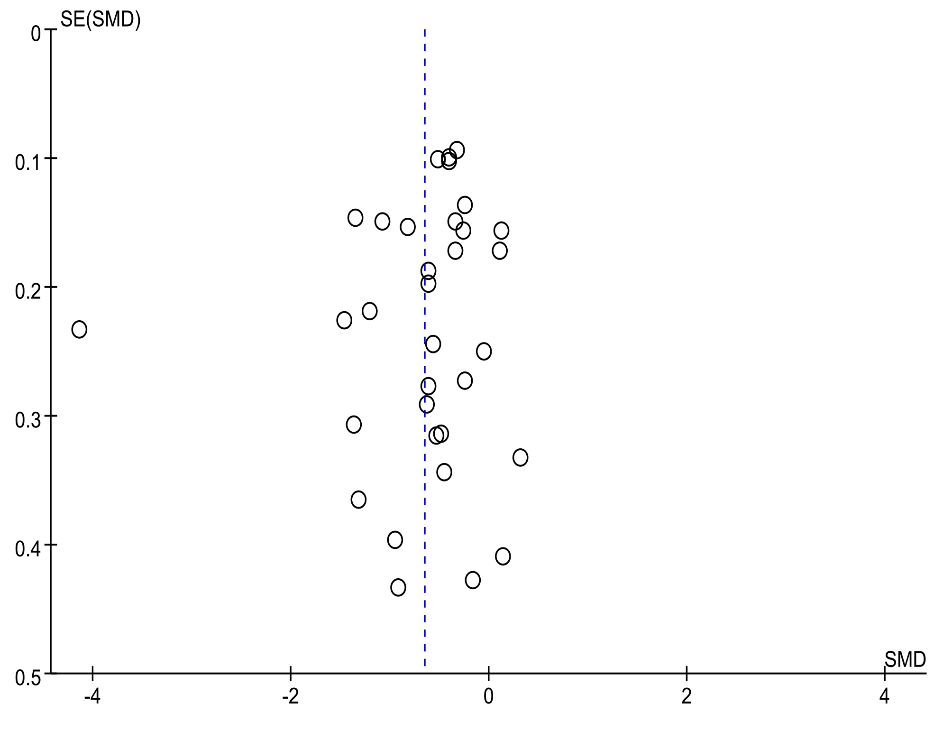
**

S4.2 Egger's test

| **Egger Regression Test for the Assessment of Publication Bias** | | | |  |  |  |
| --- | --- | --- | --- | --- | --- | --- |
| **SMD** | **Coefficient** | ***SE*** | ***t*** | ***p*** | **95%CI** | |
| Slope | -0.296 | 0.259 | -1.14 | 0.262 | -0.823 | 0.232 |
| Bias | -1.656 | 1.458 | -1.14 | 0.265 | -4.629 | 1.317 |

Appendix S5. Sensitivity Analysis by the Leave-One-Out Method

**
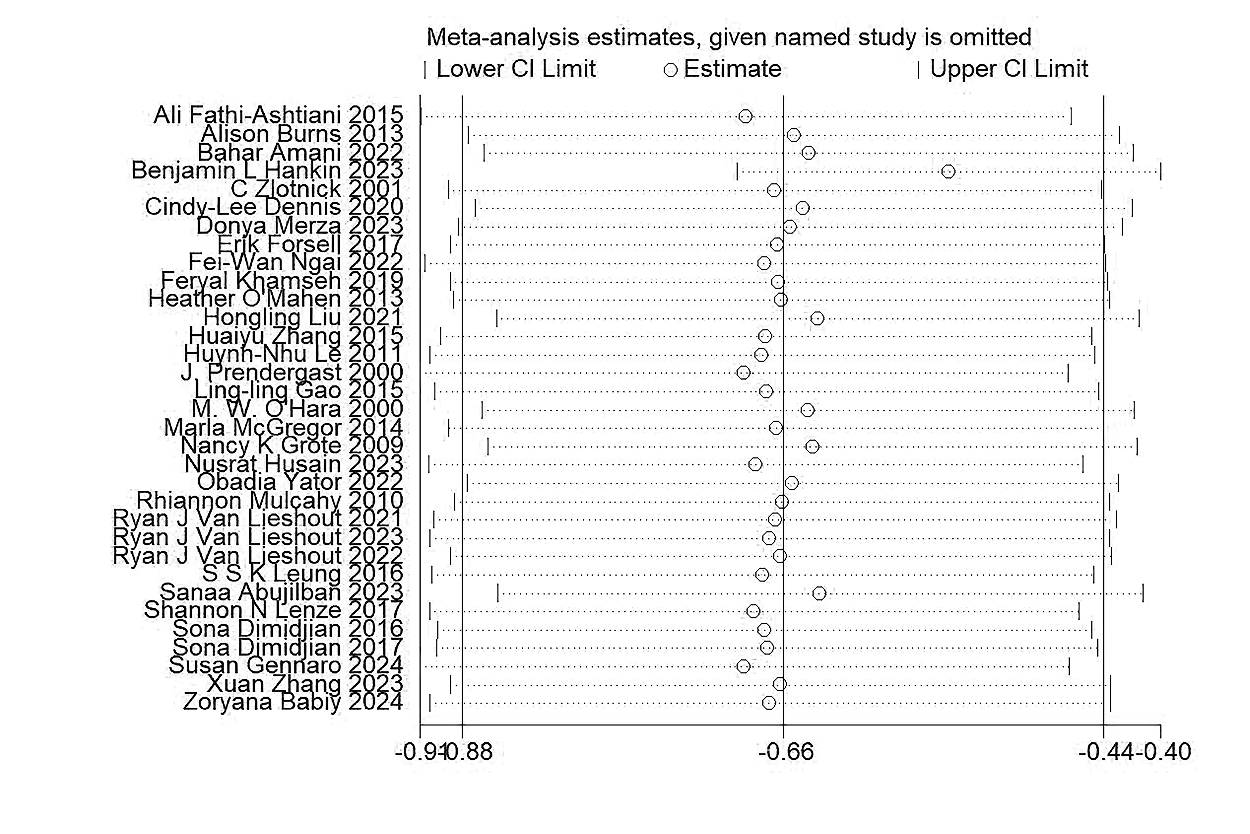
**

Appendix S6. Results of a meta-analysis of psychotherapy subgroups on perinatal depression

S6.1 Analysis type


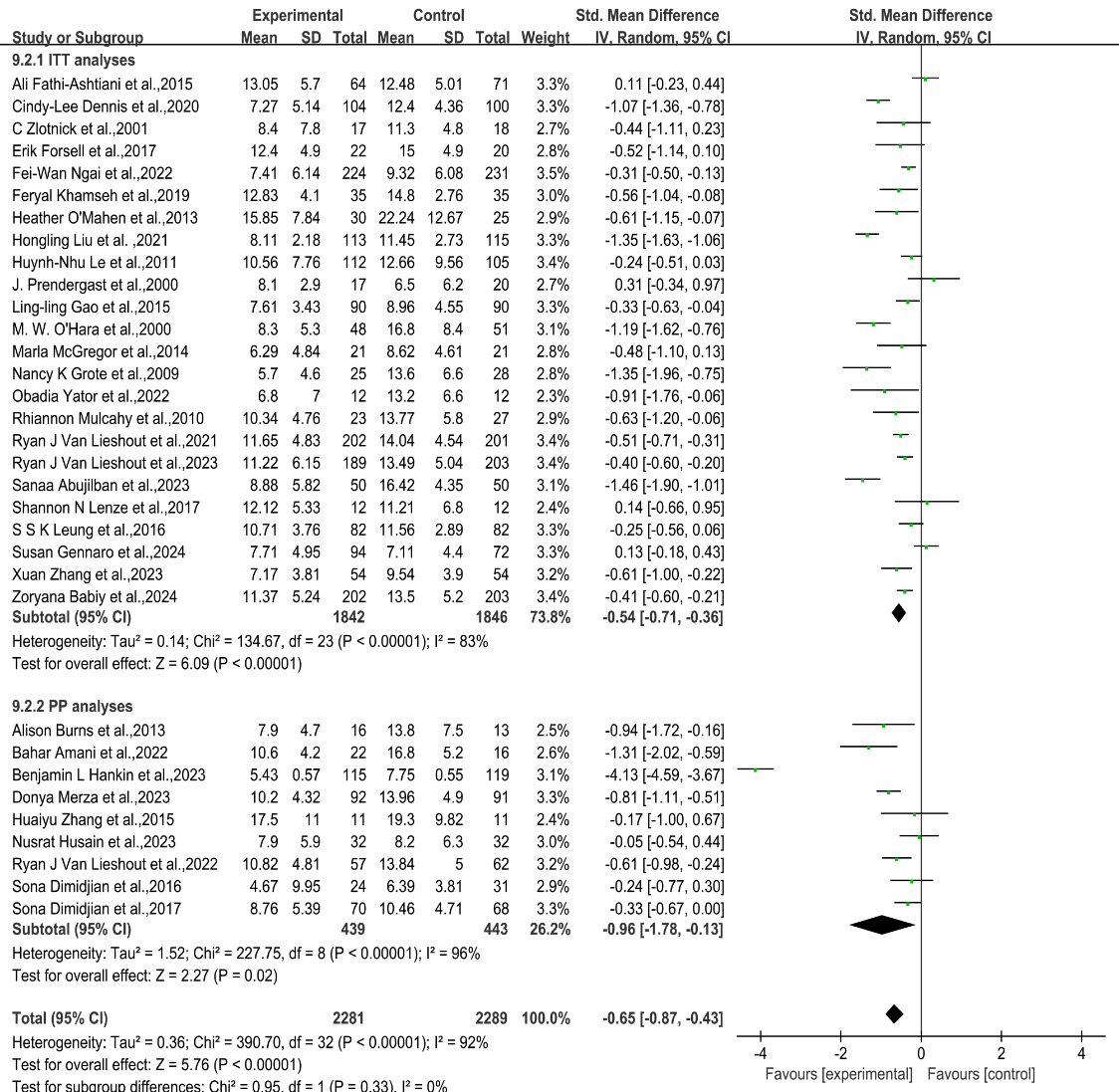


S6.2 ROB

**
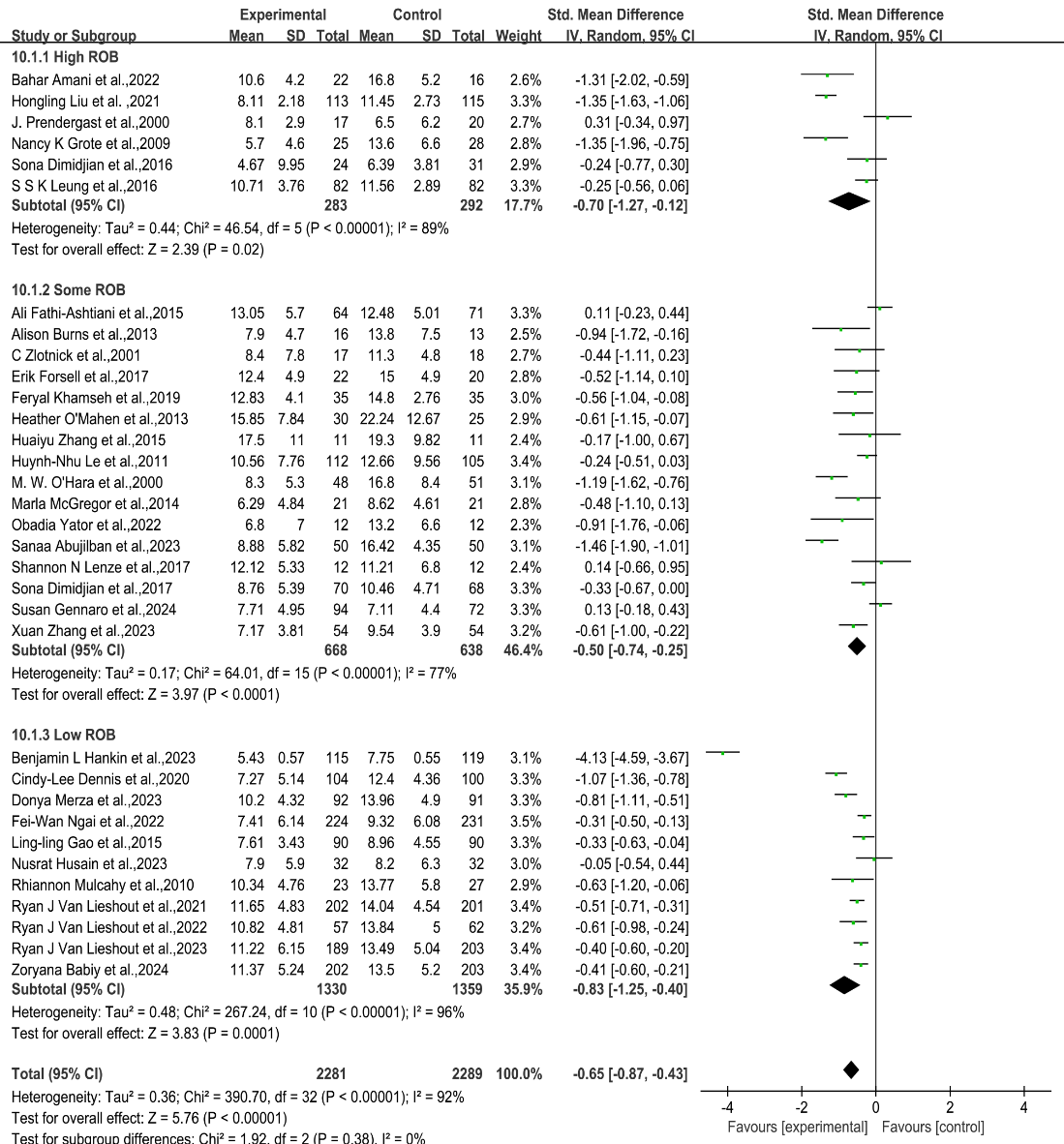
**

S6.3 Intervention duration

**
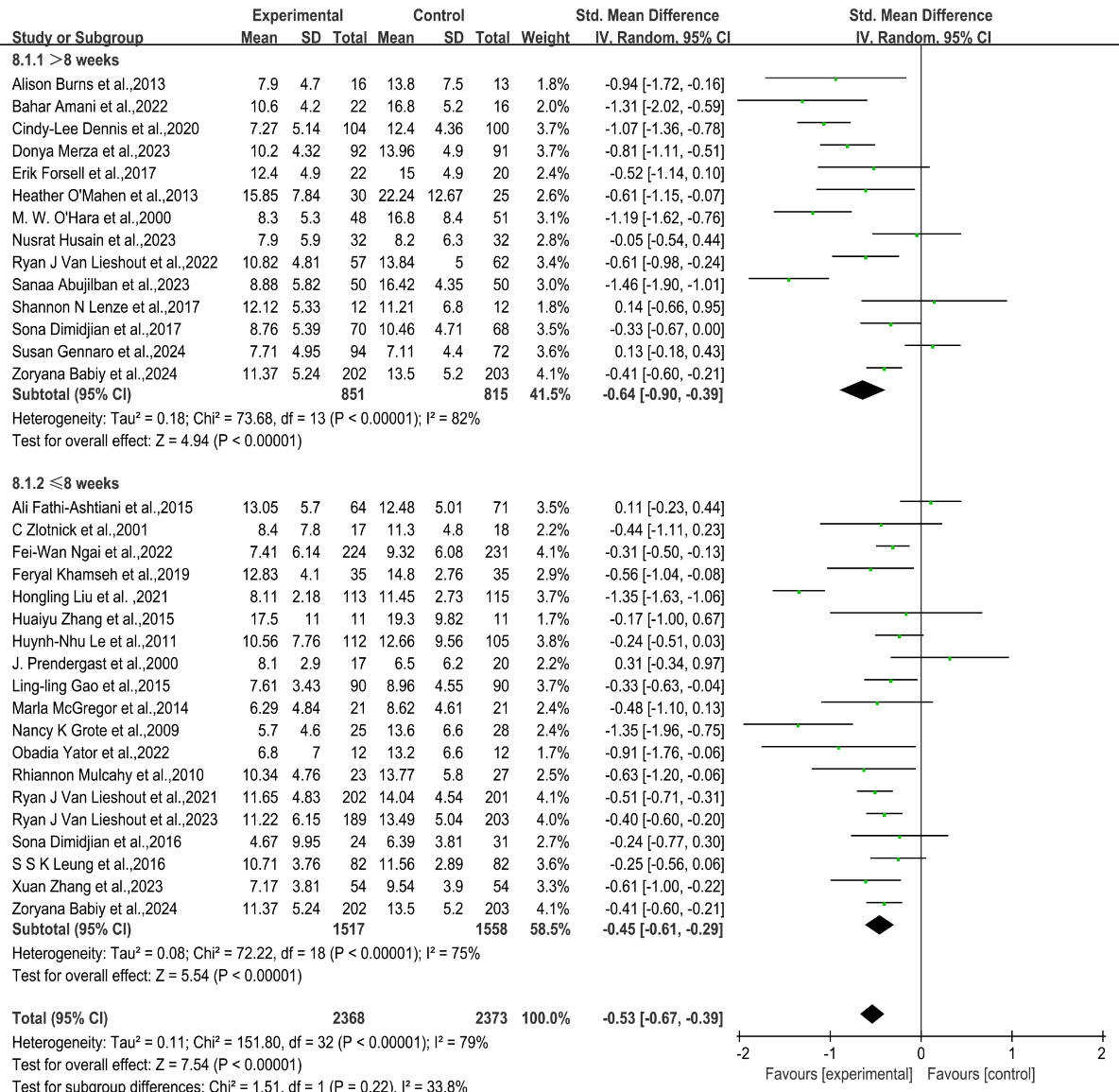
**

S6.4 Mean age

**
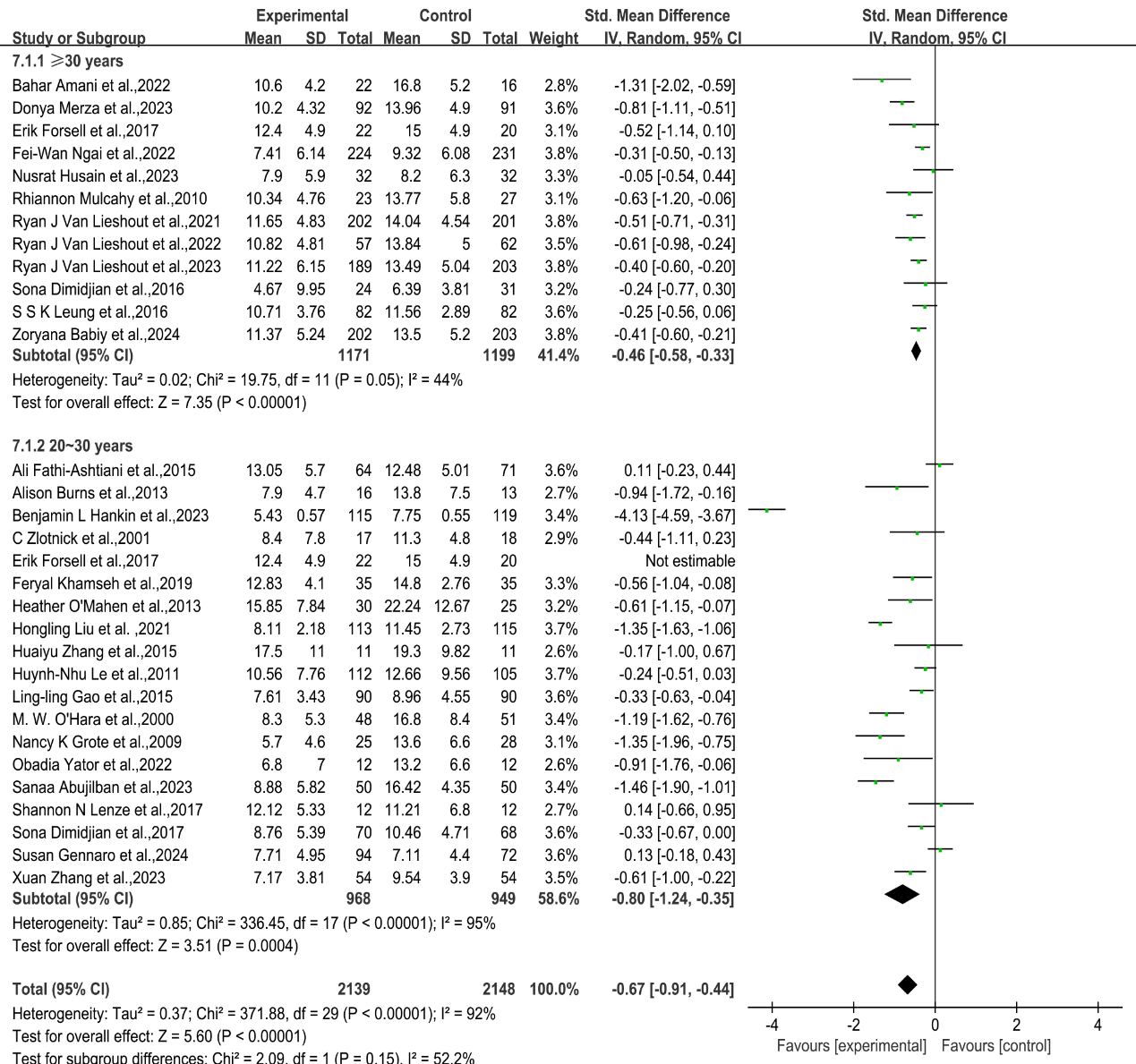
**

S6.5 Standard for evaluation

**
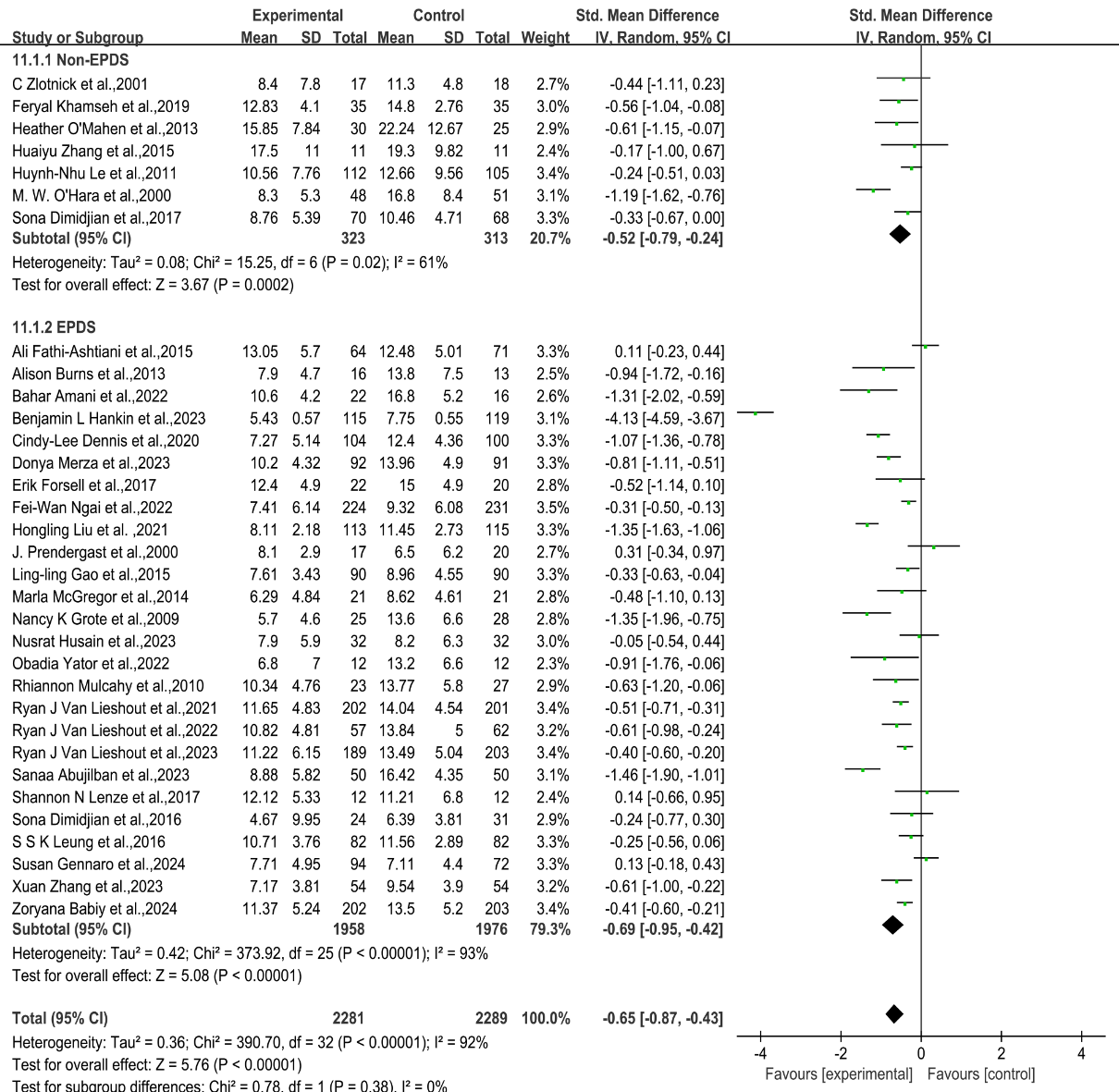
**

S6.6 Type of income

**
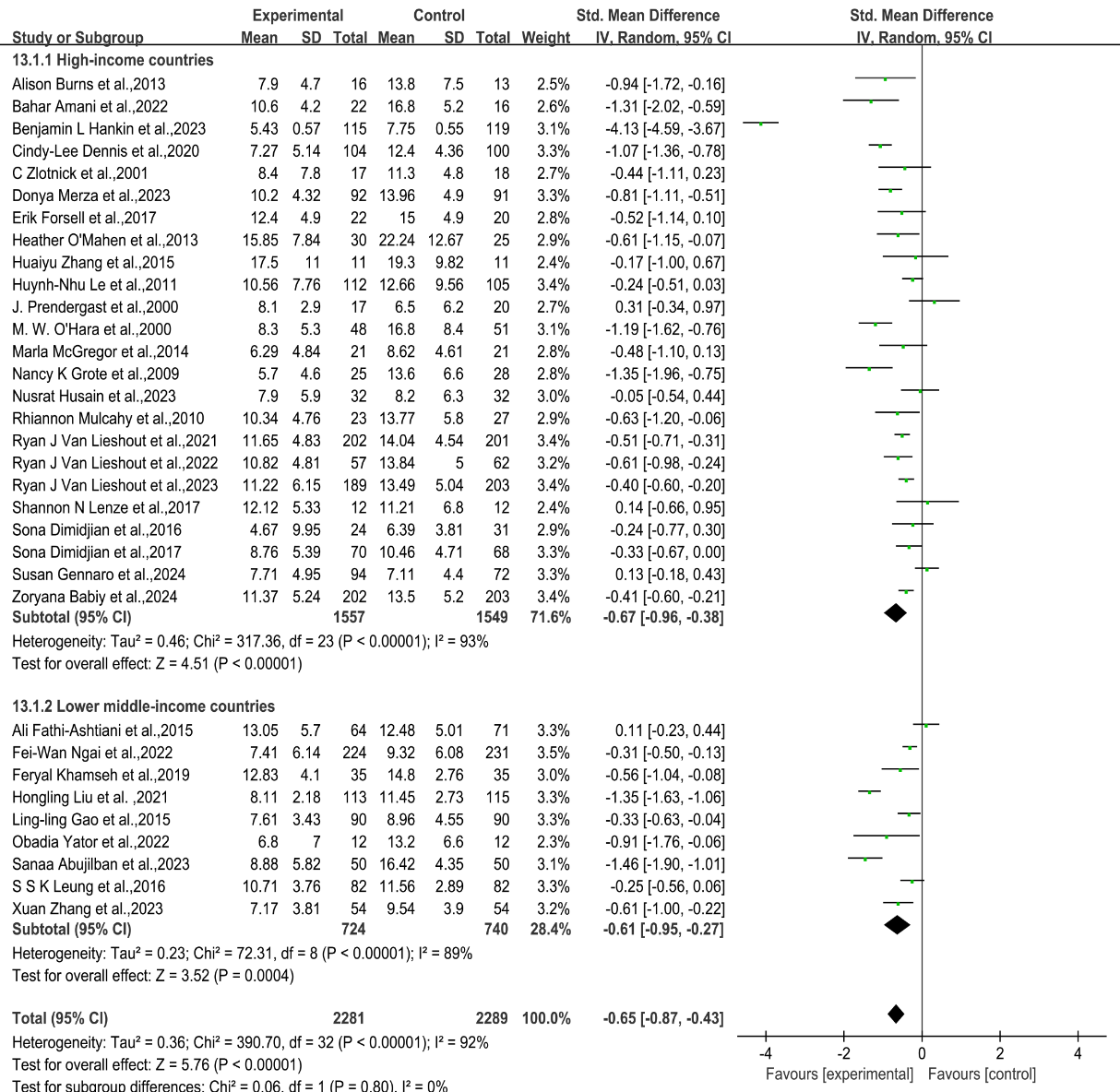
**

S6.7 Type of intervention

**
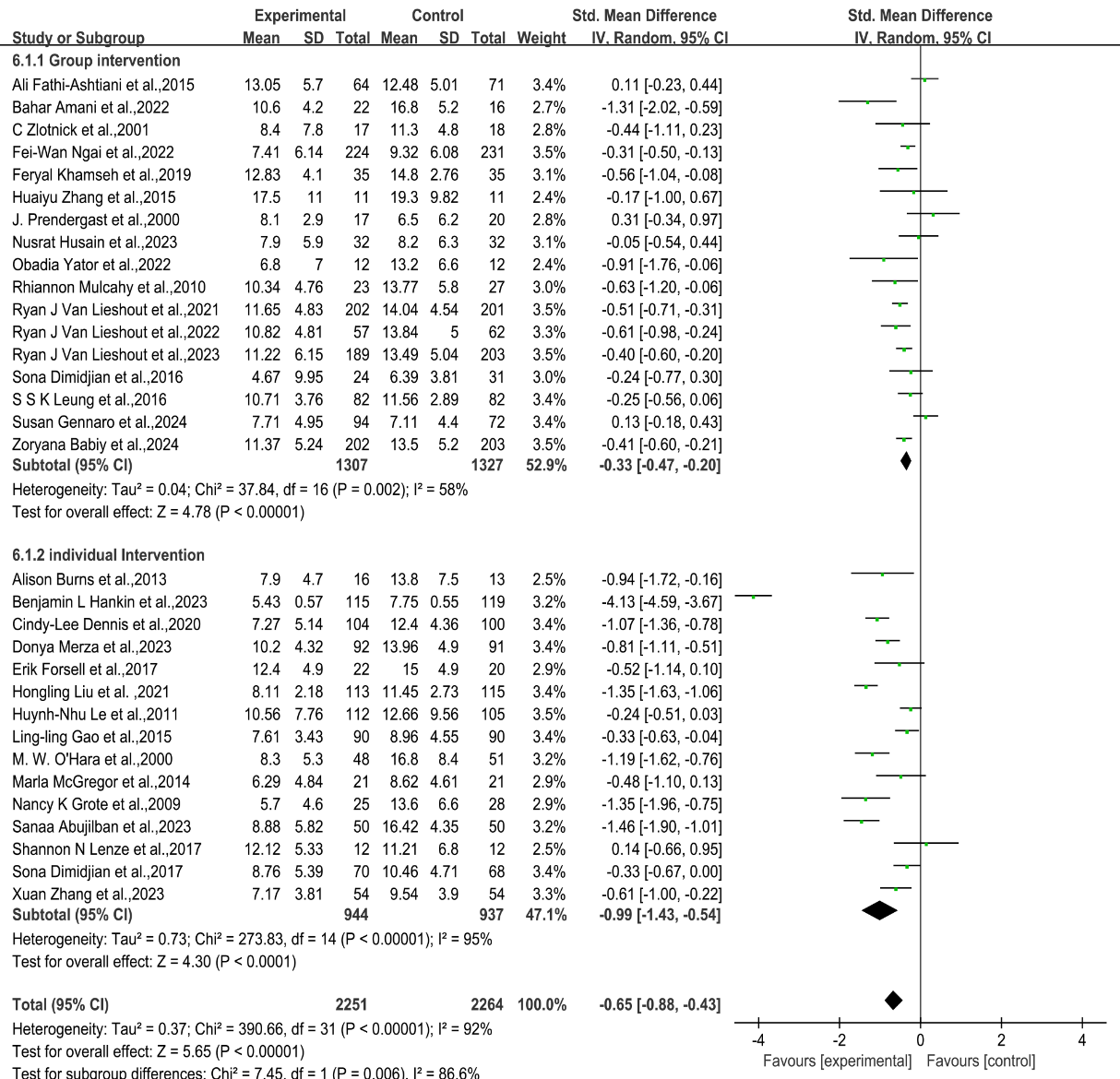
**

S6.8 Intervening period

**
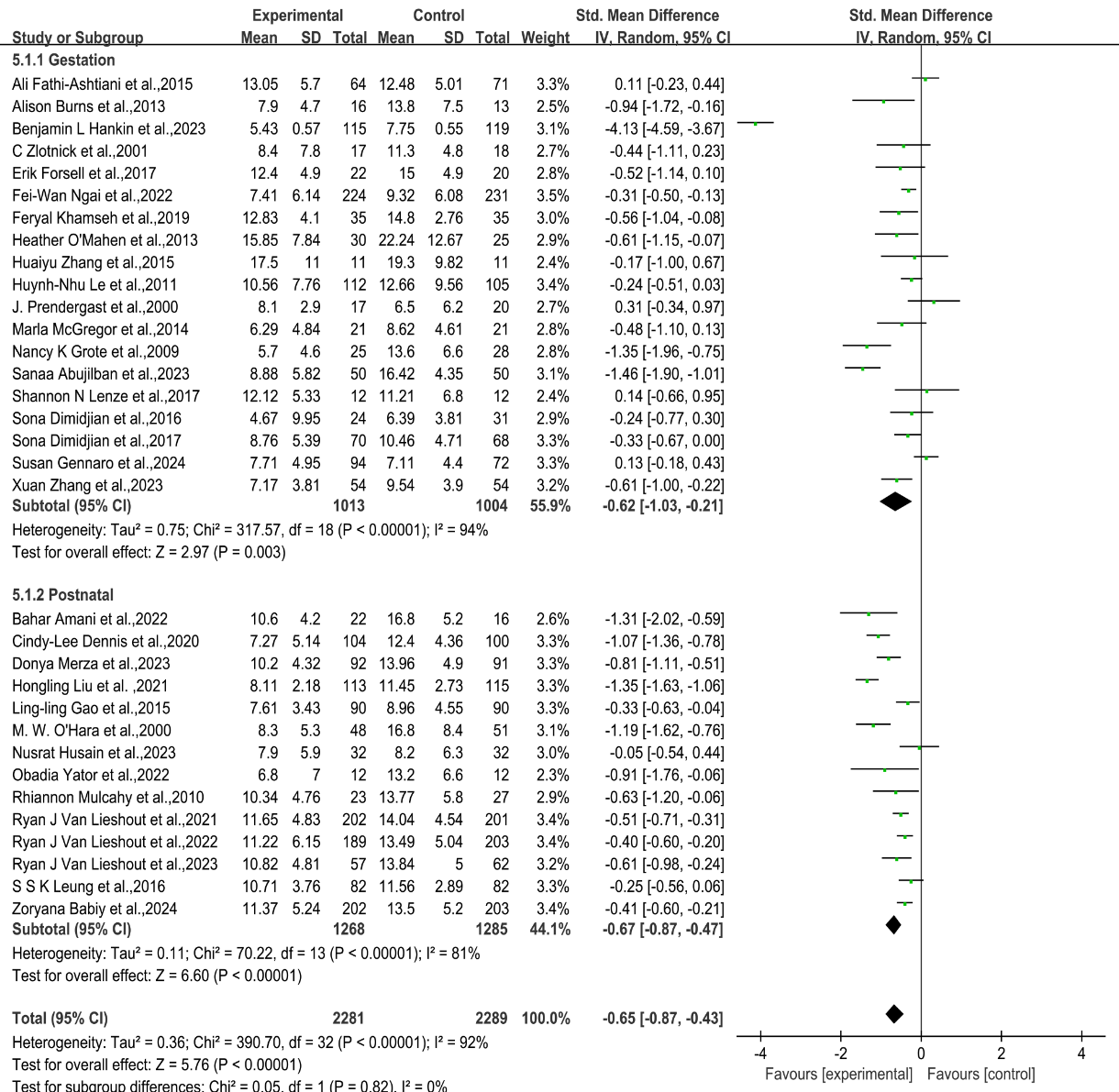
**

S6.9 Setting

**
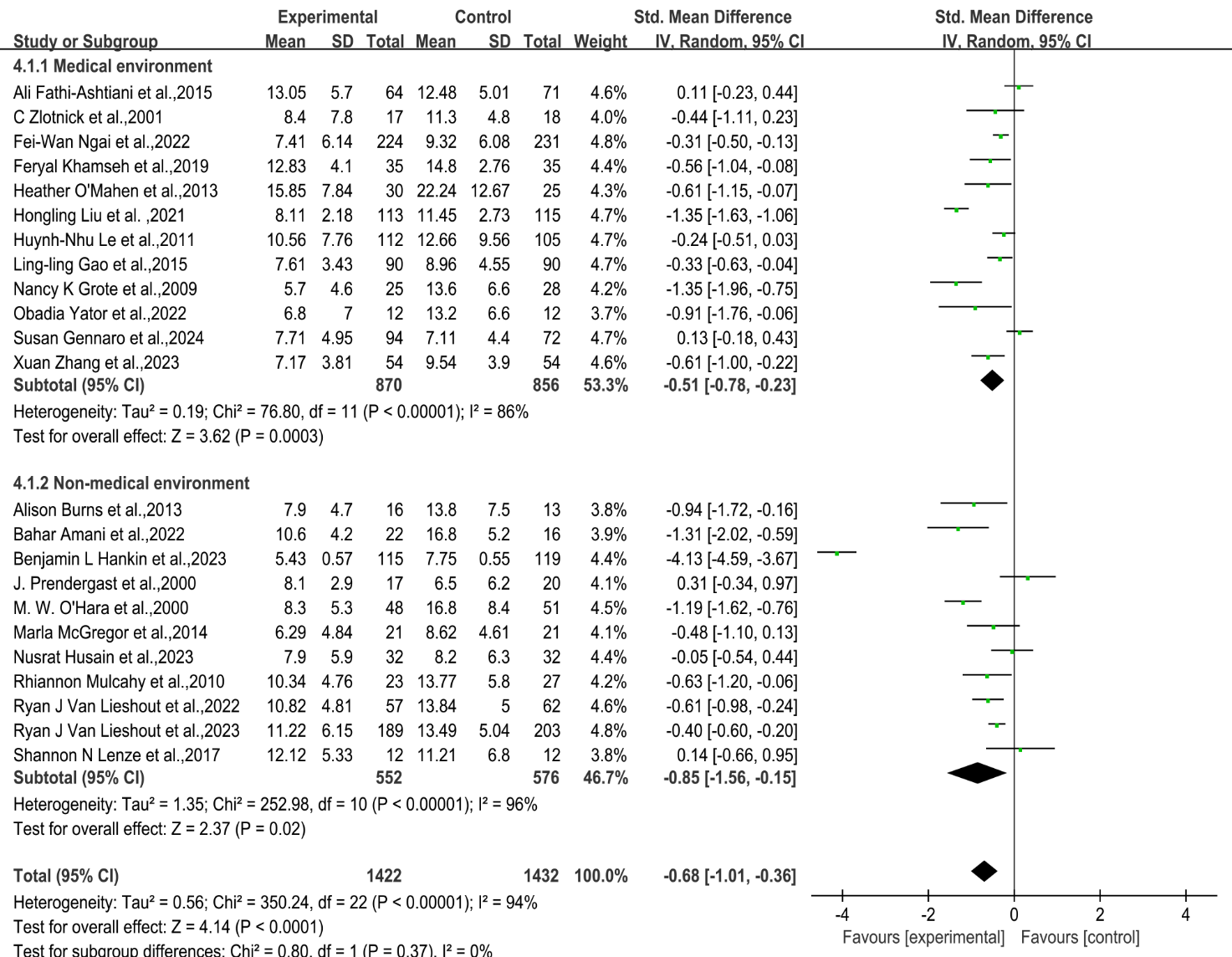
**

S6.10 Professional support

**
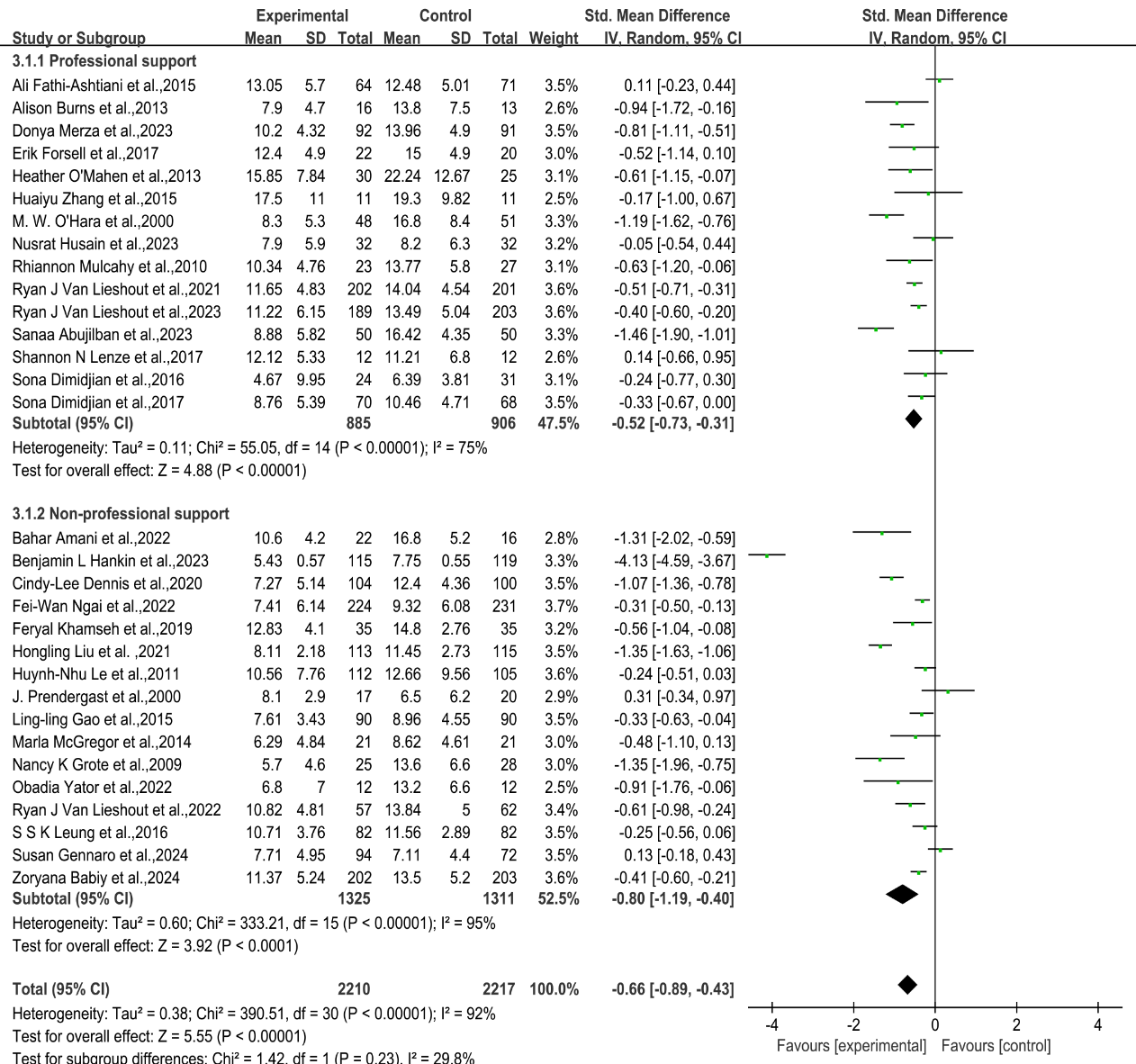
**

S6.11 Way of intervention

**
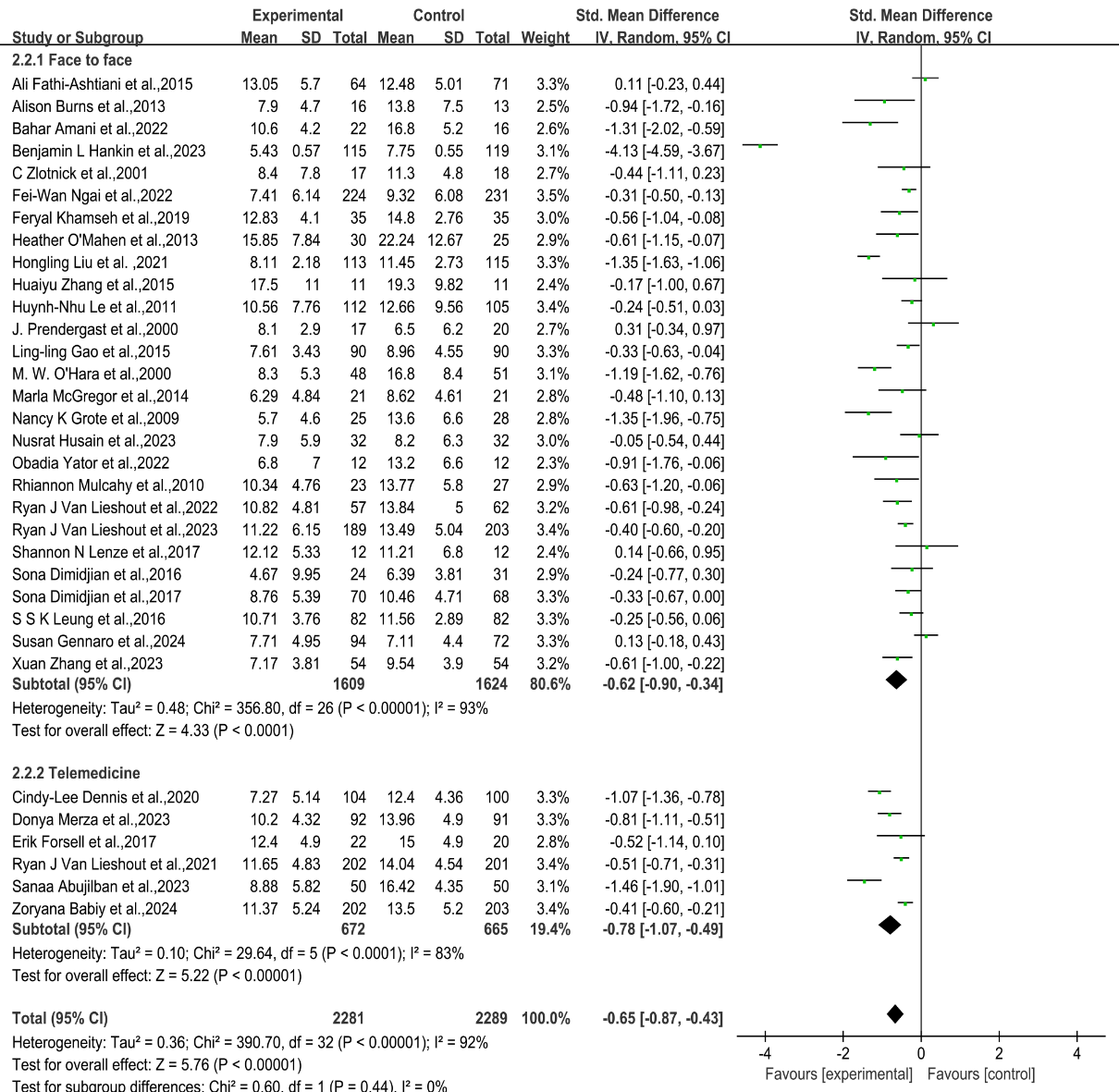
**

S6.12 Intervention category

**
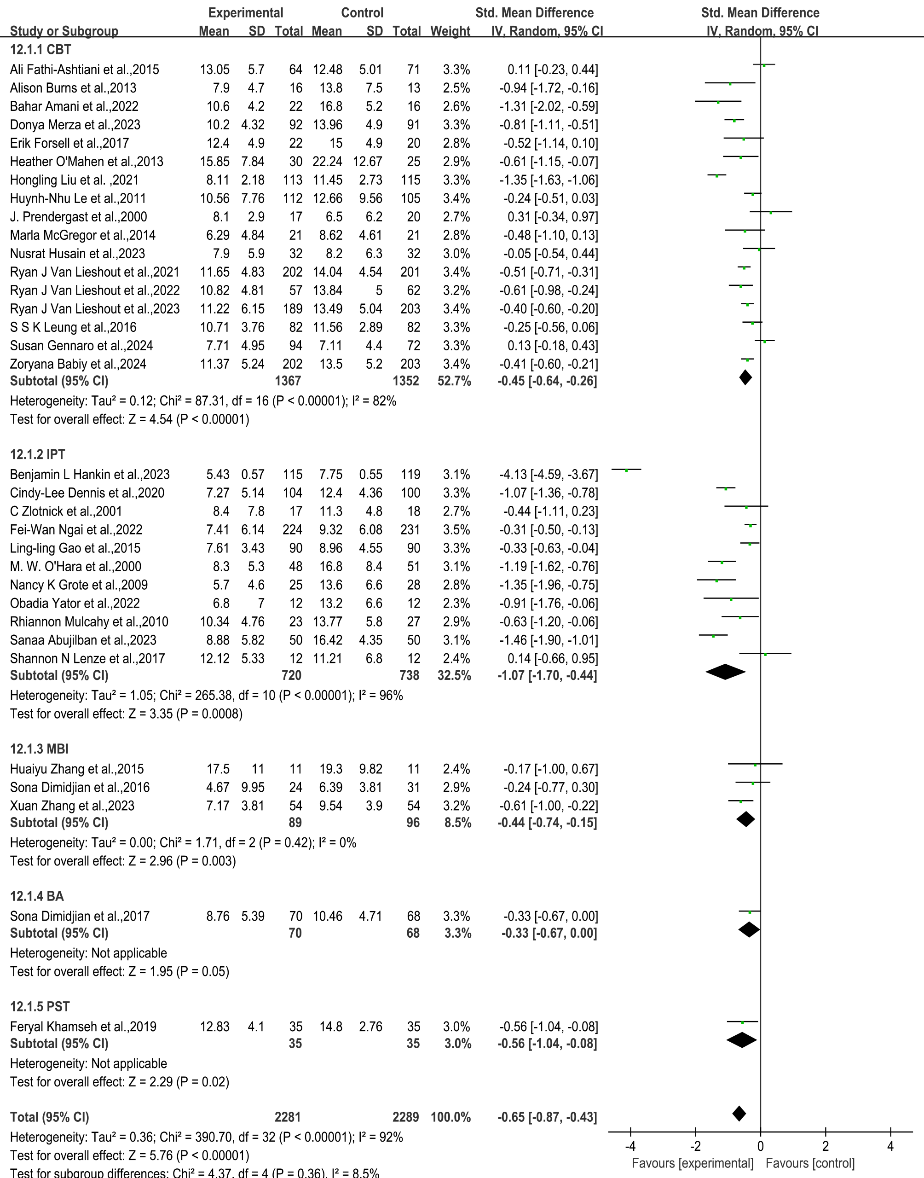
**

Appendix S7. meta-regression analysis

S7.1 Covariates for Meta-Regression

| **Variables** | **Definitions** |
| --- | --- |
| Mean age | 20~30years |
|  | ＞30years |
|  | mix |
| Setting | Medical |
|  | Non-medical |
| Way of intervention | face-to-face |
|  | Telemedicine |
| Intervening period | gestation |
|  | postnatal |
| Intervention duration | ＜8weeks |
|  | ≥8weeks |
| Type of income | Lower middle-income countries |
|  | High-income countries |
| Standard for evaluation | EPDS |
|  | Non-EPDS |
| Professional support | Professional support |
|  | non-Professional support |
| Intervention category | CBT |
|  | IPT |
|  | MBI |
|  | BA |
|  | PST |
| Year of publication | 2000-2010s |
|  | 2021-2020s |
|  | ≥2021s |
| Type of intervention | Group intervention |
|  | Individual intervention |
| ROB | High ROB |
|  | Some ROB |
|  | Low ROB |
| Analysis type | ITT analysis |
|  | PP analysis |

S7.2 Summary table of regression analysis: predictive variables and overall study β coefficients

| **Variable** | **Beta coefficient (95% CI)** | **p-value** |
| --- | --- | --- |
| Mean age | 0.062(0.007, 0.117) | **0.027** |
| Setting | -0.052(-0.642, 0.538) | 0.864 |
| Way of intervention | 0.128(-0.416, 0.671) | 0.648 |
| Intervening period | -0.109(-0.63, 0.413) | 0.681 |
| Type of intervention | -0.674(-1.224, -0.124) | **0.019** |
| Intervention duration | 0.041(0.004, 0.078) | **0.031** |
| Type of income | 0.086(-0.492, 0.665) | 0.774 |
| Standard for evaluation | -0.114(-0.626, 0.398) | 0.658 |
| Professional support | -0.142(-0.636, 0.353) | 0.574 |
| Intervention category | 0.183(-0.393, 0.759) | 0.537 |
| Year of publication | -0.211(-0.85, 0.428) | 0.519 |
| ROB | 0.187(-0.593, 0.968) | 0.638 |
| Analysis type | -1.054(-1.88, -0.227) | 0.015 |

Appendix S8. Details of interventions

**Question:** Psychological interventions compared to control groups for Perinatal depression

| **Certainty assessment** | | | | | | | **№ of patients** | | **Effect** | | **Certainty** | **Importance** |
| --- | --- | --- | --- | --- | --- | --- | --- | --- | --- | --- | --- | --- |
| **№ of studies** | **Study design** | **Risk of bias** | **Inconsistency** | **Indirectness** | **Imprecision** | **Other considerations** | **Psychological interventions** | **control groups** | **Relative (95% CI)** | **Absolute (95% CI)** |  |  |
| **Psychological intervention vs. conventional treatment** | | | | | | | | | | | | |
| 33 | randomised trials | serious^a^ | not serious | not serious | not serious | none | 2281 | 2289 | - | SMD **0.65 lower** (0.87 lower to 0.43 lower) | ⨁⨁⨁◯ Moderate^a^ | IMPORTANT |

**CI:** confidence interval; **SMD:** standardised mean difference

#### Explanations

a. Perinatal women are a special population and therefore most of the literature included is not blinded.

Appendix S9. Details of interventions

| **Study** | **Intervention** | **Treatment (detail)** |
| --- | --- | --- |
| Susan Gennaro et al.2024 | Cognitive behavioral therapy | The manualized COPE-P intervention was adapted from the evidence-based Creating Opportunities for Personal Empowerment (COPE) CBT-based programs, designed to be culturally sensitive to this population of pregnant Black and Hispanic women, and embedded into routine prenatal care. |
|  | Perinatal education | received 6 sessions of relevant patient education related to pregnancy derived from ACOG pamphlets and designed to take the same amount of time in GPC (approximately 30 minutes) as COPE-P. |
| Xuan Zhang et al.2023 | mindfulness-based intervention | The mindfulness courses included mindfulness-based stress reduction and some components of mindfulness-based cognitive therapy and acceptance and commitment therapy. |
|  | health education | The HE participants also received a 20-minute face-to face pre-intervention introduction to HE courses |
| Nusrat Husain et al.2023 | Cognitive behavioral therapy | Sobia Khan developed the Positive Health Program (PHP) as part of a PhD project and the intervention is based on CBT principles |
|  | Treatment as usual | Treatment as usual (TAU) consisted of routine assessment and management as usually conducted by general practices. |
| Ryan J Van Lieshout et al.2023 | Cognitive behavioral therapy | Each interactive workshop was run from 0900 to 1600 and consisted of four modules containing didactic teaching, group exercises/discussion, and role plays. |
|  | Treatment as usual | Could involve care from a physician, nurse practitioner, and/or midwife, as well as pharmacotherapy and/or psychotherapy from a provincially funded program. They could also utilize the services of any private therapist. |
| Benjamin L Hankin et al.2023 | Interpersonal Psychotherapy | Brief IPT consists of eight 50-minute individual sessions, approximately a week apart, with the active phase of treatment during pregnancy; there_x005f after, maintenance care is allowed with less frequent sessions. |
|  | Enhanced usual care | EUC augmented the usual standard of care pregnant individuals received within OB/GYN clinics through their obstetric clinicians and/or social workers. EUC consisted of maternity support services, which provides mental health counseling integrated within the obstetric setting and 1-on-1 consultation session with doctoral-level clinicians. Through maternity social services, pregnant individuals were offered mental health support, based on patient preferences, including prepartum depression care and other community services. |
| Ryan J Van Lieshout et al.2022 | Cognitive behavioral therapy | The first half of each session consisted of core CBT content (e.g., cognitive restructuring), and the second half included psychoeducation and/or a discussion of topics relevant to PPD (e.g., sleep, supports) and co-led by mothers and PHNs. |
|  | Treatment as usual | could involve medications and/or psychotherapy from a physician and/or clinician at a provincially-funded facility/program. Private therapists or any other treatments could also be utilized. |
| Obadia Yator et al.2022 | Interpersonal Psychotherapy | Marital conflict was attended to by inviting the male partner to attend couples’ sessions to learn problem-solving skills and were then referred to the PMTCT nurse for continual support. |
|  | Waitlist | included services for sexual and reproductive health, male partner involvement, child and maternal nutrition, and child immunization. |
| Bahar Amani et al.2022 | Cognitive behavioral therapy | The first half of each session involved instruction and practice of core CBT skills, followed by 1 hour of unstructured discussion on topics relevant to those with PPD (eg, sleep, supports).40 Core cognitive skills (e.g., cognitive restructuring) are introduced and practiced from week 1. Behavioral techniques (behavioral activation, relaxation, goal-setting) are introduced in week 2 and continue throughout. |
|  | Waitlist | could receive usual care (e.g., medication, psychotherapy) |
| Fei-Wan Ngai et al.2022 | Interpersonal Psychotherapy | The intervention consisted of three weekly 2-hour in-person antenatal sessions and two 30-minute telephone follow-up sessions delivered within 4 weeks postpartum. |
|  | Treatment as usual | received the standard prenatal and postnatal care. |
| Hongling Liu et al. 2021 | Cognitive behavioral therapy | In the intervention group, in addition to routine post partum care, participants received a 6-week cognitive behavior intervention, once a week, 1 h each time. Cognitive behavior intervention was composed of different parts. |
|  | Treatment as usual | involved the registration in com munity, postpartum life and dietary guidance provided by the hospital and community, guidance for women in maternal and newborn care, perineal care, breastfeeding, changing diapers, newborn bathing, and umbilical care, and answering questions from patients and families. |
| Feryal Khamseh et al.2019 | problem‑solving training | given routine prenatal care trainings at clinic along with problem‑solving skills training based on IDEAL model.(Identify, Define, Explore, Act, Look back) |
|  | Treatment as usual | received routine prenatal care trainings. |
| Shannon N Lenze et al.2017 | Interpersonal Psychotherapy | The clinicians participated in structured didactics and readings directed by the PI and received individual supervision using video recordings on a complete brief-IPT case prior to the study. All brief-IPT sessions were video recorded for use in supervision. |
|  | Enhanced usual care | including specialty mental health. brief case management, diapers and other baby supplies were provided. Telephone assessments were conducted every 2 weeks to assess depressive and anxiety symptoms and encourage or facilitate depression treatment. |
| Sona Dimidjian et al.2017 | Behavioral activation | included 2 days of in-person workshops and self-paced reading followed by ongoing weekly group telephonic supervision (90 min) and individual supervision as needed (30 min). |
|  | Treatment as usual | consisted of receiving routine care and completing study assessments. If study assessment indicated an elevation in depression symptom severity, then the participant and the obstetric provider were notified, and referral to a behavioral health provider at the treatment site was provided as appropriate. |
| S S K Leung et al.2016 | Cognitive behavioral therapy | The CBT intervention aimed to change cognitions and subsequently reinforce coping skills to enhance psychological resources and responses. CBT-guided participants to proactively respond to stress by reducing their negative thoughts. |
|  | Treatment as usual | provided with a booklet that contained comprehensive information and education material about perinatal depression and a list of community resources. |
| Sona Dimidjian et al.2016 | Mindfulness-Based Cognitive Therapy | The 8-session protocol for MBCT was based on the standard MBCT treatment manual. |
|  | Treatment as usual | were free to continue or initiate mental health care (as were those in the MBCT-PD condition). Tele-phone or in person at the time of assessment when their depressive symptom levels were  elevated. |
| Huaiyu Zhang et al.2015 | Mindfulness-Based Therapies | The protocol of the Mindful Motherhood intervention was adapted from empirically supported mind-body interventions, including mindfulness-based stress reduction, mindfulness-based cognitive therapy, acceptance and commitment therapy, and dialectical behavior therapy. |
|  | Treatment as usual | routine care as usual |
| Ali Fathi-Ashtiani et al.2015 | Cognitive behavioral therapy | consisted of training in self‑monitoring, self‑focused attention, relaxation, understanding of the problem, and setting up an alternative view of the problem, revising automatic thoughts, behavioral approach tasks, and exposure to worry cues. |
|  | Treatment as usual | received routine antenatal care. |
| Ling-ling Gao et al.2015 | Interpersonal Psychotherapy | The intervention was consisted of a 1-h education session before discharge and one telephone follow-up within the 2 weeks after discharge from the hospital. Specific IPT techniques, such as information giving, use of affect, clarification, signaling what is significant, reviewing relationship and communication patterns, and providing social support were applied throughout the programmed. |
|  | Treatment as usual | received a brief visit from a nurse in the postnatal ward to give them a pamphlet on sources of assistance for mothers on discharge from hospital. Women in the study group received the pamphlet and the IPT oriented postnatal psychoeducation pro_x005f gramme. |
| Marla McGregor et al.2014 | Cognitive behavioral therapy | six sessions of CBT lasting 10 min delivered by the physician providing obstetrical care. |
|  | Treatment as usual | standard prenatal care. |
| Heather O'Mahen et al.2013 | Cognitive behavioral therapy | consisted of up to twelve 50-min individual sessions of CBT, adapted for the perinatal period. |
|  | Treatment as usual | regular care. |
| Alison Burns et al.2013 | Cognitive behavioral therapy | consisted of up to 12 individual sessions of CBT at the woman’s home unless a preference was expressed to be seen elsewhere. |
|  | Treatment as usual | receive usual care from their midwife and GP. |
| Huynh-Nhu Le et al.2011 | Cognitive behavioral therapy | Specific cultural adaptations were made in the structure and content of the MB course based upon formative data gathered from focus groups with staff and clients to elucidate the unique needs of the predominantly Central American families served by the community health center. |
|  | usual care | routine prenatal care |
| Rhiannon Mulcahy et al.2010 | Interpersonal Psychotherapy | The intervention is based on Interpersonal Psycho_x005f therapy, modified for a group setting (IPT-G). Adaptations of IPT for groups include use of the processes of ‘modelling’ and ‘social reinforcement’ by group members as well as group brainstorming. |
|  | Treatment as usual | all of the options for support, assistance and treatment for postnatal depression currently being accessed by women in the Australian Capital Territory (ACT) community. |
| Nancy K Grote et al.2009 | Interpersonal Psychotherapy | a multicomponent model of care consisting of an engagement session, acute IPT-B, and maintenance IPT. |
|  | Enhanced usual care | regular care and given written educational materials about depression. |
| C Zlotnick et al.2001 | Interpersonal Psychotherapy | The first session consisted of a rationale for the program and psycho education on “baby blues” and postpartum depression. The second session focused on identifying role transitions, changes associated with role transitions, and goals for successfully managing role transitions, with an emphasis on transition to motherhood. The third session was concerned with setting goals, developing supports, and identifying potential interpersonal conflicts, especially once the baby was born. The fourth session taught skills for resolving interpersonal conflicts and reviewed the main themes of the intervention. |
|  | Treatment as usual | receive the standard medical attention and treatment that is provided to all patients attending the prenatal clinic. |
| J. Prendergast et al.2000 | Cognitive behavioral therapy | The basis of the training was a CBT-based work-book that had been prepared for the study. The work-book contained detailed psychoeducation, cognitive monitoring and thought challenging diaries and modules on anxiety management, assertiveness training, self-esteem and pleasant-event scheduling. |
|  | Treatment as usual | ideal standard care. |
| M. W. O'Hara et al.2000 | Interpersonal Psychotherapy | The initial sessions were concerned with identifying depression as a medical disorder afflicting the patient, placing the depression in an interpersonal context, reviewing the patient's current and past interpersonal relationships, and relating problematic aspects of these relationships to the patient's depression.18 Finally, the therapist and patient collaboratively identified the IPT problem area(s) most related to the episode and set treatment goals.. |
|  | Waitlist | Although no therapy was provided during this time, clinical assessments using the HRSD were conducted by telephone at 4, 8, and 12 weeks after assignment to the WLC group. Brief telephone contacts also were made at 2, 6, and 10 weeks to evaluate the patient's suicide risk and ability to wait for treatment. |
| Zoryana Babiy et al.2024 | Cognitive behavioral therapy | The intervention was a 1-day-long online CBT-based inter active workshop that ran 9:00 a.m. to 4:00 p.m. and contained a total of 6 h of instruction (two 15-min and one 30-min break was provided). |
|  | Treatment as usual | include any medications, psychotherapy, and other treatments participants wished to use. |
| Donya Merza et al.2023 | Cognitive behavioral therapy | Led by two randomly selected peer facilitators via the online video conference. The first half of each session involved teaching and practice of core CBT skills, fol_x005f lowed by 1-h of unstructured discussion on topics relevant to those with PPD. |
|  | Treatment as usual | Involve any type of treatment deemed to have been required by the participant. |
| Sanaa Abujilban et al.2023 | Interpersonal Psychotherapy | Seven sessions (each half an hour) of telephone-based IPT were offered twice weekly to those assigned to the intervention arm: one pretherapy orientation, five intermediates, and one closing session |
|  | Treatment as usual | regular care |
| Ryan J Van Lieshout et al.2021 | Cognitive behavioral therapy | The intervention was a 1-day (9 AM to 4 PM) interactive workshop consisting of didactic teaching, group exercises/ discussion, and role playing in 4 modules（reviewed PPD etiology、 focused on cognitive skills、built behavioral skills、provided an opportunity for goal setting and action planning）. |
|  | Treatment as usual | involve medications and/or psychotherapy from a physician and/or clinician at a provincially funded facility/program. Private therapists or any other treatments could also be used. |
| Cindy-Lee Dennis et al.2020 | Interpersonal Psychotherapy | Women allocated to the intervention group received 12 weekly 60 min telephone-IPT sessions delivered by a trained nurse, with the first contact to initiate treatment occurring within 72 h of trial enrolment. |
|  | Treatment as usual | standard postpartum care |
| Erik Forsell et al.2017 | internet-delivered cognitive-behavioral therapy | a semi-structured telephone interview that primarily consisted of the depression segment of the SCID-I interview and everything but the depression segment from the M.I.N.I. interview (version 6; Sheehan et al., 1998) to cover potential co-morbidities. |
|  | Treatment as usual | continuation of their current maternity care for 10 weeks |

Appendix S9. Rational of excluding studies during the full-text screening

| **No** | **Title** | **Reason for exclusion** |
| --- | --- | --- |
| 1 | Aromatherapy in postpartum depression: A clinical trial based on precede model | Non-English |
| 2 | Effect of cognitive-behavioral group therapy on gestational depression: A clinical trial | Non-English |
| 3 | Effectiveness of stress management training on stress reduction in pregnant women | Non-English |
| 4 | Effectiveness of cognitive-behavioral stress management intervention on anxiety and depression during pregnancy | Non-English |
| 5 | Evaluating the effect of progressive muscle relaxation training with guided imagery on the severity of depressive symptoms in postpartum period | Non-English |
| 6 | Combined cognitive behavior therapy with systematic family therapy in patients with mild to moderate postpartum depression | Non-English |
| 7 | Effect of cognitive therapy based on positive thinking on depression, stress, and anxiety of mothers after recovering from COVID-19 | Non-English |
| 8 | The effects of music therapy on postpartum blues and maternal attachment of puerperal women | Non-English |
| 9 | A pilot randomized controlled trial of cognitive behavioural therapy for women with antenatal depression: Infant temperament and sleep | Meeting abstract |
| 10 | Development of a CBT program for depression during pregnancy-beating the blues before birth | Meeting abstract |
| 11 | CBT for low income perinatal women | Meeting abstract |
| 12 | A multi-site randomized controlled trial to evaluate the effect of telephone-based interpersonal psychotherapy by trained nurses for the treatment of postpartum depression | Meeting abstract |
| 13 | Psychological treatment of antenatal depression and anxiety: The effects on both mother and child | Meeting abstract |
| 14 | Cognitive behavioral therapy for treatment of antenatal anxiety and depressive symptoms: A randomized controlled trial | Meeting abstract |
| 15 | Web-based cognitive behavioural therapy for postnatal depression | Meeting abstract |
| 16 | A randomized controlled trial of internet based cognitive behavioural therapy (CBT) versus treatment as usual for pregnant women with high levels of depression at queen charlotte's hospital | Meeting abstract |
| 17 | How might mindfulness based cognitive therapy help prevent perinatal depression: The role of mindfulness, decentering, and rumination | Meeting abstract |
| 18 | Primary care social worker administered psychotherapy for postpartum depression | Meeting abstract |
| 19 | Anticipate: A pilot randomised trial of CBT for antenatal depression and validation of depression screening by midwives | Meeting abstract |
| 20 | Mindfulness based cognitive therapy for the prevention of perinatal depression: Initial findings | Meeting abstract |
| 21 | Mindfulness based prevention of perinatal depression: Innovative treatment development | Meeting abstract |
| 22 | Multidisciplinary model of nurse midwife administered psychotherapy for postpartum depression | Meeting abstract |
| 23 | A clinical trial of an adapted treatment of CBT with depressed mothers in home visitation | Meeting abstract |
| 24 | 995 The impact of art therapy in women at risk for postpartum depression: a pilot study | Meeting abstract |
| 25 | Shape of change in internet based behavioral activation treatment for depression | Secondary analysis |
| 26 | One-Day Workshop Beneficial for Postpartum Depression | Review |
| 27 | Perinatal mental health and COVID-19 in Japan | Review |
| 28 | Cognitive behavior therapy for postpartum depression | Review |
| 29 | Telehealth and women's perinatal mental health | Review |
| 30 | Perinatal e-mental health support: evidence and challenges in translation to practice | Review |
| 31 | Problem solving strategy can help prevent depression in low income mothers | Review |
| 32 | Collaborative decision making improves interpersonal psychotherapy efficiency: A randomized clinical trial with postpartum women | Irrelevant interventions or comparisons |
| 33 | Antenatal mobile-delivered mindfulness-based intervention to reduce perinatal depression risk and improve obstetric and neonatal outcomes: A randomized controlled trial | Irrelevant interventions or comparisons |
| 34 | Smartphone-assisted online brief cognitive behavioral therapy to treat maternal depression: findings of a randomized controlled trial | Irrelevant interventions or comparisons |
| 35 | Antenatal depression: Efficacy of a pre-post therapy study and repercussions in motor development of children during the first 18 months postpartum. Study: "Pregnancy care, healthy baby" | Irrelevant interventions or comparisons |
| 36 | Scaling Up Maternal Mental healthcare by Increasing access to Treatment (SUMMIT) through non-specialist providers and telemedicine: a study protocol for a non-inferiority randomized controlled trial | Irrelevant interventions or comparisons |
| 37 | Influence of adjuvant metacognitive detached mindfulness and stress management training compared to pharmacologic treatment in primiparae with postpartum depression | Irrelevant interventions or comparisons |
| 38 | An open trial of mindfulness-based cognitive therapy for the prevention of perinatal depressive relapse/recurrence | Irrelevant interventions or comparisons |
| 39 | A controlled clinical treatment trial of interpersonal psychotherapy for depressed pregnant women at 3 New York City sites | Irrelevant interventions or comparisons |
| 40 | Effectiveness of a Brief Psychological Intervention Delivered by Nurse for Depression in Pregnancy: Study Protocol for a Multicentric Randomized Controlled Trial from India | Irrelevant interventions or comparisons |
| 41 | A proof-of-concept pilot randomized comparative trial of brief internet-based compassionate mind training and cognitive-behavioral therapy for perinatal and intending to become pregnant women | Irrelevant interventions or comparisons |
| 42 | Task-sharing of psychological treatment for antenatal depression in Khayelitsha, South Africa: Effects on antenatal and postnatal outcomes in an individual randomised controlled trial | Irrelevant interventions or comparisons |
| 43 | Does attending community music interventions lead to changes in wider musical behaviours? The effect of mother-infant singing classes on musical behaviours amongst mothers with symptoms of postnatal depression | Irrelevant interventions or comparisons |
| 44 | The effect of progressive muscle relaxation and guided imagery on stress, anxiety, and depression of pregnant women referred to health centers | Irrelevant interventions or comparisons |
| 45 | Effect of singing interventions on symptoms of postnatal depression: Three-arm randomised controlled trial | Irrelevant interventions or comparisons |
| 46 | Efficacy of an internet-based cognitive behavioral stress management training in women with idiopathic preterm labor: A randomized controlled intervention study | Irrelevant interventions or comparisons |
| 47 | Efficacy of a maternal depression prevention strategy in Head Start: A randomized clinical trial | Irrelevant interventions or comparisons |
| 48 | Prenatal listening to songs composed for pregnancy and symptoms of anxiety and depression: a pilot study | Irrelevant interventions or comparisons |
| 49 | Group interpersonal psychotherapy for postnatal depression: a pilot study | Irrelevant interventions or comparisons |
| 50 | Prenatal depression: a randomized controlled trial in the emotional health of primiparous women | Irrelevant interventions or comparisons |
| 51 | Effects of a prenatal mindfulness program on longitudinal changes in stress, anxiety, depression, and mother-infant bonding of women with a tendency to perinatal mood and anxiety disorder: a randomized controlled trial | Irrelevant interventions or comparisons |
| 52 | Multicentre randomised controlled trial of a group psychological intervention for postnatal depression in British mothers of South Asian origin (ROSHNI-2): Study protocol | Irrelevant interventions or comparisons |
| 53 | A mindfulness and compassion-based program applied to pregnant women and their partners to decrease depression symptoms during pregnancy and postpartum: study protocol for a randomized controlled trial | Irrelevant interventions or comparisons |
| 54 | Preventing perinatal depression in Spain: a pilot evaluation of Mamas y Bebes | Irrelevant interventions or comparisons |
| 55 | Assessing the effectiveness of mindfulness-based programs on mental health during pregnancy and early motherhood - A randomized control trial | Irrelevant interventions or comparisons |
| 56 | The Effectiveness and Cost-Effectiveness of Web-Based and Home-Based Postnatal Psychoeducational Interventions for First-Time Mothers: Randomized Controlled Trial Protocol | Irrelevant interventions or comparisons |
| 57 | The effects of mindfulness training compared to pregnancy support on maternal stress and depression: A pilot randomised trial | Irrelevant interventions or comparisons |
| 58 | The Effect of Bergamot Essential Oil Aromatherapy on Improving Depressive Mood and Sleep Quality in Postpartum Women: A Randomized Controlled Trial | Irrelevant interventions or comparisons |
| 59 | A Group Videoconference Intervention for Reducing Perinatal Depressive Symptoms: A Telehealth Pilot Study | Irrelevant interventions or comparisons |
| 60 | The preventive effect of internet-based cognitive behavioral therapy for prevention of depression during pregnancy and in the postpartum period (iPDP): a large scale randomized controlled trial | Irrelevant interventions or comparisons |
| 61 | The effect of telephone-based cognitive-behavioral therapy on postnatal depression: A randomized controlled trial | Irrelevant interventions or comparisons |
| 62 | A Randomised Controlled Trial of Therapist-Assisted, Internet-Delivered Cognitive Behavior Therapy for Women with Maternal Depression | Irrelevant interventions or comparisons |
| 63 | A peer-facilitated psychological group intervention for perinatal women living with HIV and depression in Tanzania-Healthy Options: A cluster-randomized controlled trial | Irrelevant interventions or comparisons |
| 64 | A cognitive-behavioral intervention for postpartum anxiety and depression: Individual phone vs. group format | Irrelevant interventions or comparisons |
| 65 | The Role of Engagement in Mindfulness-Based Cognitive Therapy for the Prevention of Depressive Relapse/Recurrence in Perinatal Women | Irrelevant interventions or comparisons |
| 66 | Effects of mindfulness on maternal stress, depressive symptoms and awareness of present moment experience: A pilot randomised trial | Irrelevant interventions or comparisons |
| 67 | Proof of concept: Partner-Assisted Interpersonal Psychotherapy for perinatal depression | Irrelevant interventions or comparisons |
| 68 | Exposure-based cognitive-behaviour therapy for anxiety-related disorders in pregnancy (ADEPT): Results of a feasibility randomised controlled trial of time-intensive versus weekly CBT | Irrelevant interventions or comparisons |
| 69 | Couple-based interpersonal psychotherapy for first-time parents: A process evaluation | Non-primary outcome |
| 70 | Cognitive behaviour therapy-based intervention by community health workers for mothers with depression and their infants in rural Pakistan: A cluster-randomised controlled trial | Non-primary outcome |
| 71 | The effect of a telephone-based cognitive behavioral therapy on quality of life: a randomized controlled trial | Non-primary outcome |
| 72 | Telephone-based cognitive-behavioral therapy on postnatal depression and quality of life | Non-primary outcome |
| 73 | Early intervention to prevent adverse child emotional and behavioural development following maternal depression in pregnancy: study protocol for a randomised controlled trial | Non-primary outcome |
| 74 | A spiritual intervention to reduce stress, anxiety and depression in pregnant women: Randomized controlled trial | Non-primary outcome |
| 75 | The effects of a music and singing intervention during pregnancy on maternal well-being and mother-infant bonding: a randomised, controlled study | Non-primary outcome |
| 76 | 80: Effects of cognitive behavioural therapy for antenatal anxiety and depression on mother and offspring | Non-primary outcome |
| 77 | The efect of an adapted Mindfulness_x005fBased Stress Reduction program on mental health, maternal bonding and birth outcomes in psychosocially vulnerable pregnant women: a study protocol for a randomized controlled trial in a Danish hospital-based outpatient setting | Non-primary outcome |
| 78 | Effects of Perinatal Cognitive Behavioral Therapy on Delivery Mode, Fetal Outcome, and Postpartum Depression and Anxiety in Women | Non-primary outcome |
| 79 | App-based intervention for reducing depressive symptoms in postpartum women: Protocol for a feasibility randomized controlled trial | App or webpage intervention |
| 80 | Feasibility and Acceptability of a Mindfulness-Based Smartphone App among Pregnant Women with Obesity | App or webpage intervention |
| 81 | Feasibility and impact of a mental health chatbot on postpartum mental health: a randomized controlled trial | App or webpage intervention |
| 82 | Using an Electronic Mindfulness-based Intervention (eMBI) to improve maternal mental health during pregnancy: Results from a randomized controlled trial | App or webpage intervention |
| 83 | App-based intervention for reducing depressive symptoms in postpartum women: Protocol for a feasibility randomized controlled trial | App or webpage intervention |
| 84 | Trial of a patient-directed eHealth program to ameliorate perinatal depression: the MomMoodBooster2 practical effectiveness study | App or webpage intervention |
| 85 | The Efficacy of Be a Mom, a Web-Based Intervention to Prevent Postpartum Depression: Examining Mechanisms of Change in a Randomized Controlled Trial | App or webpage intervention |
| 86 | Effectiveness and cost-effectiveness of an electronic mindfulness-based intervention to improve maternal mental health in the peripartum: study protocol for a randomised controlled trial | App or webpage intervention |
| 87 | Cost-effectiveness of Web-Based and Home-Based Postnatal Psychoeducational Interventions for First-time Mothers: Economic Evaluation Alongside Randomized Controlled Trial | App or webpage intervention |
| 88 | Preventing Postpartum Depression in the Early Postpartum Period Using an App-Based Cognitive Behavioral Therapy Program: A Pilot Randomized Controlled Study | App or webpage intervention |
| 89 | Feasibility of a Web-Based Intervention to Prevent Perinatal Depression and Promote Human Milk Feeding: Randomized Pilot Trial | App or webpage intervention |
| 90 | Be a Mom's efficacy in enhancing positive mental health among postpartum women presenting low risk for postpartum depression: Results from a pilot randomised trial | App or webpage intervention |
| 91 | Cost-utility of a web-based intervention to promote maternal mental health among postpartum women presenting low risk for postpartum depression | App or webpage intervention |
| 92 | HAPPY MAMA Project (Part 2). Maternal Distress and Self-Efficacy: A Pilot Randomized Controlled Field Trial | App or webpage intervention |
| 93 | Web-based treatment for depression in pregnancy: A feasibility study of Mum2BMoodBooster | App or webpage intervention |
| 94 | Trial of a patient-directed eHealth program to ameliorate perinatal depression: the MomMoodBooster2 practical effectiveness study | App or webpage intervention |
| 95 | Feasibility and Acceptability of Internet-Based Interpersonal Psychotherapy for Stress, Anxiety, and Depression in Prenatal Women: Thematic Analysis | App or webpage intervention |
| 96 | A smartphone-assisted brief online cognitive-behavioral intervention for pregnant women with depression: a study protocol of a randomized controlled trial | App or webpage intervention |
| 97 | Effectiveness of Smartphone-Based Mindfulness Training on Maternal Perinatal Depression: Randomized Controlled Trial | App or webpage intervention |
| 98 | Testing the Benefits of Reducing Prenatal Maternal Depression for Maternal and Infant outcomes: A randomized controlled trial | App or webpage intervention |
| 99 | Healthy Moms and Babies Preventive Psychological Intervention Application: A Study Protocol | App or webpage intervention |
| 100 | Internet-based behavioural activation to improve depressive symptoms and prevent child abuse in postnatal women (SmartMama): a protocol for a pragmatic randomized controlled trial | App or webpage intervention |
| 101 | Be a mom, a web-based intervention to promote positive mental health among postpartum women with low risk for postpartum depression: Exploring psychological mechanisms of change | App or webpage intervention |
| 102 | Internet-based cognitive-behavioural therapy for prevention of depression during pregnancy and in the post partum (iPDP): a protocol for a large-scale randomised controlled trial | App or webpage intervention |
| 103 | Effectiveness and cost-effectiveness of an electronic mindfulness-based intervention (eMBI) on maternal mental health during pregnancy: the mindmom study protocol for a randomized controlled clinical trial | App or webpage intervention |
| 104 | Be a Mom's Efficacy in Enhancing Positive Mental Health among Postpartum Women Presenting Low Risk for Postpartum Depression: Results from a Pilot Randomized Trial | App or webpage intervention |
| 105 | Internet cognitive behavioural therapy for women with postnatal depression: A randomized controlled trial of MumMoodBooster | App or webpage intervention |
| 106 | Effectiveness of an app-based cognitive behavioral therapy program for postpartum depression in primary care: A randomized controlled trial | App or webpage intervention |
| 107 | The effectiveness of a guided internet-based tool for the treatment of depression and anxiety in pregnancy (MamaKits Online): Randomized controlled trial | App or webpage intervention |
| 108 | Preventing Postpartum Depression With Mindful Self-Compassion Intervention: A Randomized Control Study | App or webpage intervention |
| 109 | Effects of a Brief Electronic Mindfulness-Based Intervention on Relieving Prenatal Depression and Anxiety in Hospitalized High-Risk Pregnant Women: Exploratory Pilot Study | App or webpage intervention |
| 110 | Be a Mom, a web-based intervention to prevent postpartum depression: Results from a pilot randomized controlled trial | App or webpage intervention |
| 111 | A Blended Cognitive-Behavioral Intervention for the Treatment of Postpartum Depression: Study Protocol for a Randomized Controlled Trial | App or webpage intervention |
| 112 | A mobile health mindfulness intervention for women with moderate to moderately severe postpartum depressive symptoms: Feasibility study | App or webpage intervention |
| 113 | Internet and Face-to-face Cognitive Behavioral Therapy for Postnatal Depression Compared With Treatment as Usual: Randomized Controlled Trial of MumMoodBooster | App or webpage intervention |
| 114 | A study protocol of mobile phone app-based cognitive behaviour training for the prevention of postpartum depression among high-risk mothers | App or webpage intervention |
| 115 | Mediation analyses of Internet-facilitated cognitive behavioral intervention for maternal depression | App or webpage intervention |
| 116 | A randomized controlled trial of 'MUMentum Pregnancy': Internet-delivered cognitive behavioral therapy program for antenatal anxiety and depression | App or webpage intervention |
| 117 | A randomised controlled trial of 'MUMentum postnatal': Internet-delivered cognitive behavioural therapy for anxiety and depression in postpartum women | App or webpage intervention |
| 118 | Regaining 'MUMentum': randomized controlled trial of online CBT for perinatal distress, anxiety, and depression | App or webpage intervention |
| 119 | Mamma Mia - A randomized controlled trial of an internet-based intervention for perinatal depression | App or webpage intervention |
| 120 | Be a mom, a web-based intervention to prevent postpartum depression: The enhancement of self-regulatory skills and its association with postpartum depressive symptoms | App or webpage intervention |
| 121 | A Group-Based Online Intervention to Prevent Postpartum Depression (Sunnyside): Feasibility Randomized Controlled Trial | App or webpage intervention |
| 122 | Move my mood: Development and evaluation of a mobile mental health self-help app using behavioral activation for women with postpartum depression | App or webpage intervention |
| 123 | Internet-based cognitive behavioural therapy (iCBT) for perinatal anxiety and depression versus treatment as usual: Study protocol for two randomised controlled trials | App or webpage intervention |
| 124 | Strongest FamiliesTM Managing Our Mood (MOM): A randomized controlled trial of a distance intervention for women with postpartum depression | App or webpage intervention |
| 125 | Mom-net: Evaluation of an internet-facilitated cognitive behavioral intervention for low-income depressed mothers | App or webpage intervention |
| 126 | An open trial of web-based mindfulness-based cognitive therapy for perinatal women at risk for depressive relapse | App or webpage intervention |
| 127 | Internet cognitive behavioral therapy for women with postnatal depression: A randomized controlled trial of MumMoodBooster | App or webpage intervention |
| 128 | Development and assessment of a mobile phone-based intervention to reduce maternal depression and improve child health | App or webpage intervention |
| 129 | Mummoodbooster-an interactive internet treatment for postnatal depression | App or webpage intervention |
| 130 | A pilot, exploratory report on dyadic interpersonal psychotherapy for perinatal depression | App or webpage intervention |
| 131 | Antenatal mindfulness intervention to reduce depression, anxiety and stress: a pilot randomised controlled trial of the MindBabyBody program in an Australian tertiary maternity hospital | App or webpage intervention |
| 132 | Netmums: A phase II randomized controlled trial of a guided Internet behavioural activation treatment for postpartum depression | App or webpage intervention |
| 133 | Internet-based behavioral activation--treatment for postnatal depression (Netmums): a randomized controlled trial | App or webpage intervention |
| 134 | Momcare: Culturally relevant treatment services for perinatal depression | App or webpage intervention |
| 135 | Effectiveness of Digital Guided Self-help Mindfulness Training During Pregnancy on Maternal Psychological Distress and Infant Neuropsychological Development: Randomized Controlled Trial | App or webpage intervention |
| 136 | Internet-based cognitive therapy for women with antenatal depressive symptoms during the COVID-19 pandemic: protocol for a multi-center randomized controlled trial across China | App or webpage intervention |
| 137 | Maternal low-intensity psychosocial telemental interventions in response to COVID-19 in Qatar: study protocol for a randomized controlled trial | No original data |
| 138 | The effect of telephone-based interpersonal psychotherapy for the treatment of postpartum depression: Study protocol for a randomized controlled trial | No original data |
| 139 | Effects of mindfulness-based cognitive therapy in pregnancy on psychological distress and gestational age: Outcomes of a randomized controlled trial | No original data |
| 140 | Reducing the Risk for Postpartum Depression in Adolescent Mothers: A Randomized Controlled Trial | No original data |
| 141 | Protocol for a mechanistic study of mindfulness based cognitive therapy during pregnancy | No original data |
| 142 | Lessons learned from a pilot randomized controlled trial of dyadic interpersonal psychotherapy for perinatal depression in a low-income population | No original data |
| 143 | Mindfulness-based cognitive therapy for psychological distress in pregnancy: study protocol for a randomized controlled trial | No original data |
| 144 | A comparative study of the effects of problem-solving skills training and relaxation on the score of self-esteem in women with postpartum depression | No original data |
| 145 | A randomized controlled trial of psychological interventions for postnatal depression | No original data |
| 146 | Pregnancy stressors and postpartum symptoms of depression and anxiety: The moderating role of a cognitive-behavioural therapy (CBT) intervention | No original data |
| 147 | Effects of a prenatal mindfulness program on longitudinal changes in stress, anxiety, depression, and mother–infant bonding of women with a tendency to perinatal mood and anxiety disorder: a randomized controlled trial | No original data |
